# Formation and inhibition mechanism of novel angiotensin I converting enzyme inhibitory peptides from *Chouguiyu*

**DOI:** 10.3389/fnut.2022.920945

**Published:** 2022-07-22

**Authors:** Daqiao Yang, Laihao Li, Chunsheng Li, Shengjun Chen, Jianchao Deng, Shaoling Yang

**Affiliations:** ^1^Key Laboratory of Aquatic Product Processing, Ministry of Agriculture and Rural Affairs, National R&D Center for Aquatic Product Processing, South China Sea Fisheries Research Institute, Chinese Academy of Fishery Sciences, Guangzhou, China; ^2^Co-Innovation Center of Jiangsu Marine Bio-industry Technology, Jiangsu Ocean University, Lianyungang, China; ^3^College of Food Science and Engineering, Ocean University of China, Qingdao, China; ^4^Collaborative Innovation Center of Seafood Deep Processing, Dalian Polytechnic University, Dalian, China

**Keywords:** *Chouguiyu*, ACE inhibitory peptide, metagenomics, peptidomics, molecular docking, correlation network

## Abstract

Angiotensin I converting enzyme (ACE) inhibitory peptides from fermented foods exhibit great potential to alleviate hypertension. In this study, the peptide extract from *Chouguiyu* exhibited a good inhibition effect on ACE, and the inhibition rate was significantly enhanced after fermentation for 8 days. The ACE inhibitory peptides were further identified, followed by their inhibition and formation mechanisms using microbiome technology and molecular docking. A total of 356 ACE inhibitory peptides were predicted using *in silico*, and most ACE inhibitory peptides increased after fermentation. These peptides could be hydrolyzed from 94 kinds of precursor proteins, mainly including muscle-type creatine kinase, nebulin, and troponin I. P1 (VEIINARA), P2 (FAVMVKG), P4 (EITWSDDKK), P7 (DFDDIQK), P8 (IGDDPKF), P9 (INDDPKIL), and P10 (GVDNPGHPFI) were selected as the core ACE inhibitory peptides according to their abundance and docking energy. The salt bridge and conventional hydrogen bond connecting unsaturated oxygen atoms in the peptides contributed most to the ACE inhibition. The cleavage proteases from the microbial genera in *Chouguiyu* for preparing these 7 core ACE inhibitory peptides were further analyzed by hydrolysis prediction and Pearson's correlation. The correlation network showed that P7, P8, and P9 were mainly produced by the proteases from LAB including *Lactococcus, Enterococcus, Vagococcus, Peptostreptococcus*, and *Streptococcus*, while P1, P2, P4, and P10 were mainly Produced by *Aeromonas, Bacillus, Escherichia*, and *Psychrobacter*. This study is helpful in isolating the proteases and microbial strains to directionally produce the responding ACE inhibitory peptides.

## Introduction

Hypertension is a chronic health problem closely related to many diseases, such as stroke, heart attack, and heart failure (1). Angiotensin I converting enzyme (ACE) can elevate blood pressure by dilating the arterial blood vessels throughout the body (2). Many drugs have been designed to inhibit ACE, such as captopril and lisinopril, but synthetic drugs have many side effects (3). A large number of food-derived peptides have been identified as potential substitutes for synthetic drugs to treat hypertension (4, 5). These inhibitory peptides have distinct structural characteristics, such as possessing fewer amino acids, abundant hydrophobic amino acids, and aliphatic, aromatic, or positively charged amino acids in the N/C terminal (6, 7). These active sequences are collected on bioactive websites such as BIOPEP-UWM and AHTpin, which can predict the activity and characteristics of unknown peptides based on reliable models (6). Meanwhile, virtual screening is becoming an effective, accurate, and promising method to find novel active peptides and has been widely used in the screening of ACE inhibitory peptides (8). However, the ACE inhibitory peptides are usually prepared from protein hydrolysates using commercially available proteases. The restriction of their cleavage locations leads to the low diversity of ACE inhibitory peptides, thereby limiting the discovery of novel ACE inhibitory peptides with high bioactivity.

Fermented foods are the source of many bioactive peptides (9) and have significant advantages in the production of ACE inhibitory peptides. Plenty of fermented foods are focused on isolating ACE inhibitory peptides such as cheese (10), fermented goat milk (11), fish sauce (12), and bean paste (13). In fermented foods, the rich ACE inhibitory peptides benefit from the complex microorganisms that can produce various proteases (14). *Chouguiyu* is a typical fermented fish with rich peptides. Most research has focused on the formation mechanism of its distinctive flavor and taste (15, 16). However, few studies can discuss the role of microorganisms from *Chouguiyu* in the formation mechanism of ACE inhibitory peptides.

Microbiome technology facilitates the construction of a bridge between microorganisms and metabolites (17, 18), which can be applied to explore the formation mechanism of ACE inhibitory peptides. Shotgun metagenomics is the technology to obtain all the genetic information of microorganisms in the environment, using random fragments of microbial genes to splice small fragments into longer sequences for analysis (19). Peptidomics is an effective method to study the structure and abundance of bioactive peptides (20). With the aid of statistical methods, the metabolism formation of bioactive peptides by microorganisms can be illuminated through microbiome technology. However, there is a lack of study on the formation mechanism of bioactive peptides in fermented foods. Molecular docking, as a kind of computer-aided drug design technology, provides convenience for visualizing the activity of peptides and predicts the possible inhibition mechanisms between a small molecule ligand and a given protein receptor (21, 22). This technology has been widely used to screen ACE inhibitory peptides (23). However, the interaction at the atomic level between ACE inhibitory peptides and ACE has rarely been illustrated by molecular docking.

In this study, the microbial community and free peptides were analyzed by microbiome technology, including metagenomics and peptidomics, followed by molecular docking at the atomic level to explore the inhibition mechanism of ACE inhibitory peptides in *Chouguiyu*. The formation mechanism of ACE inhibitory peptides in *Chouguiyu* was further illustrated by the construction of a correlation network. This study is expected to illustrate the formation mechanism of the ACE inhibitory peptides in *Chouguiyu* from the perspective of microbial metabolism and to provide vital information on proteases and the related microbial strains for the production of ACE inhibitory peptides.

## Materials and methods

### Materials and chemicals

*Chouguiyu* was made from frozen mandarin fish (*Siniperca chuatsi*, av. 500 g) in Anhui, China according to the previous study (24). After fermentation at 15–20°C for 8 days, three samples of *Chouguiyu* were collected for each individual sample at 0 days (D0 group), 4 days (D4 group), and 8 days (D8 group) for further study. The reagents used for liquid chromatogram tandem mass were of mass pure grade, and all other reagents were of analytical grade.

### Preparation of peptide extract from *Chouguiyu*

Peptide extract was obtained according to the previous study (25). In brief, after being peeled, the *Chouguiyu* (50.0 g) was smashed and homogenized for 0.5 h with 200 ml hydrochloric acid (0.01 mol/L). After centrifugation at 8,000 r/min and 4°C for 20 min, the supernatant was added with triploid ethanol for 20 min at 4°C and was then centrifuged to remove protein. The supernatant was lyophilized and stored at −80°C after desalination.

### Analysis of ACE inhibitory activity of peptide extract in *Chouguiyu*

The assay of ACE inhibitory activity was performed using the ACE inhibitory assay kit (ACE kit-WST, A502, Dojindo, Kumamoto Ken, Japan) (13). In brief, the peptide extract of D0, D4, and D8 was dissolved in Milli-Q water (RephiLe Bioscience, Shanghai, China) with a concentration of 20 g/L. The sample (20 μl) was mixed with substrate buffer (20 μl) and enzyme working solution (20 μl). After incubation for 60 min at 37°C, the indicator working solution (200 μl) was added and incubated at room temperature for 10 min. Captopril was used as a positive control throughout the experiment. The absorbance of each sample was measured at 450 nm using an enzyme-labeled instrument (Sunrise-basic Tecan, Männedorf, Switzerland). The ACE inhibition rate (I) was calculated using the following equation:


(1) $$\begin{array}{l} I\;\left(\%\right)=\frac{A_{blank1}-A_{sample}}{A_{blank1}-A_{blank2}}\times 100 \end{array}$$

where *A*_blank1_ was the absorbance without sample, *A*_blank2_ was the absorbance without enzyme working solution, and *A*_sample_ was the absorbance with samples or positive control.

### Identification of peptides in *Chouguiyu* by peptidomics

The peptidomics was conducted using the ultra-high performance liquid chromatography tandem mass as described previously (26) with some modifications. In brief, the peptide extract (4 mg) was dissolved in 0.1% trifluoroacetic acid and was then desalted with the Oasis HLB column (Waters, Milford, MA, USA). The eluents were dissolved by 50% acetonitrile with 0.5% trifluoroacetic acid and were desalted with the Oasis MCX column (Waters, Milford, MA, USA). After being freeze-dried, the samples were dissolved at 10 μg/μl using 2% acetonitrile and 0.1% formic acid. The samples (10 μl) were analyzed using the Easy-nLC 1200 UPLC-Q Exactive HF-X tandem mass spectrometer (Thermo, Waltham, MA, USA), coupled with a Thermo C-18 column (Thermo, Waltham, MA, USA). The instrument was operated in a single charge mode ([M+H]^+^) at the range of 350–1,500 (m/z). The peptide sequences were identified using Proteome Discoverer^TM^ version 2.4 (Thermo, Waltham, MA, USA) according to the protein sequences of *Siniperca chuatsi*. False discovery rate <0.01, unique peptide ≥1, and Sequest HT >0 were used as the criteria to confirm the credible peptides. The peptides with ACE inhibitory activity from *Chouguiyu* were further predicted using AHTpin (http://crdd.osdd.net/raghava/ahtpin/).

### Microorganism determination by shot-gun metagenomics

The microorganisms in *Chouguiyu* were obtained after centrifugation of fermented liquid in each sample. Shot-gun metagenomics was adopted to analyze the microorganisms as in the previous study (27). In brief, the genomic DNA of microorganisms was fragmented to construct the DNA library using the NEXTFLEX® Rapid DNA-Seq (Bioo Scientific, Austin, TX, USA). The DNA library was then used for paired-end sequencing through the Illumina Hiseq Xten (Illumina Inc., San Diego, CA, USA). The abundance of microorganisms at the phylum and genus level was calculated according to the sum of all gene abundances. The genes were aligned to eggNOG database version 4.5.1 to obtain their functional annotation (28).

### *In silico* analysis of ACE inhibitory peptides

The toxicity of peptides was obtained from ToxinPred (https://webs.iiitd.edu.in/raghava/toxinpred/multi_submit.php). The isoelectric point of peptides was obtained from Expasy (https://web.expasy.org/compute_pi/). The hydrophobicity was predicted from PepDraw (http://www.tulane.edu/~biochem/WW/PepDraw/). The instability of ACE inhibitory peptides was calculated from ProtParam (https://web.expasy.org/protparam/). PepBank was used to determine whether the peptide was reported on http://pepbank.mgh.harvard.edu/search/basic, containing the searches of Pubmed, PDF, ASPD, and UniProt. The water solubility was searched using http://www.innovagen.com/proteomics-tools. The predicted enzymes of core ACE inhibitory peptides were obtained from BIOPEP-UWM (https://biochemia.uwm.edu.pl/) (29), and their categories were searched from the enzyme database (https://www.brenda-enzymes.org/).

### Molecular docking between ACE and ACE inhibitory peptides

The three-dimensional crystal structure of ACE (PDB ID: 1O8A) was downloaded from RCSB PDB (https://www.rcsb.org/) and prepared by Discovery Studio 2019 Client (San Diego, CA, USA) (30). The structures of ACE inhibitory peptides were generated using Chem Draw version 19.0 (Cambridge soft, Cambridge, MA, USA), and their energy was minimized under CHARMm using DS. The peptides were preloaded with hydrogen polar atoms, electric charges, and CFF force fields. The three-dimensional crystal structure of ACE was conducted with the clean process to delete water and former ligands, and its active sites of receptor cavities were further defined. The molecular docking between the ACE and ACE inhibitory peptides was conducted using the CDOCKER module. The hypotensive drug captopril was used as the positive control to dock with the ACE.

### Statistical analysis

The principal component analysis (PCA) and abundance heatmaps of peptides were generated using Origin 9.5C (OriginLab Corp, Northampton, VA, USA). The peptide profile was drawn using Peptigram (http://bioware.ucd.ie/peptigram) (31). The correlation heatmap between the peptides and microorganisms was carried out using R (https://www.R-project.org/). The interaction network was constructed using Gephi (32).

## Results and discussion

### Identification of ACE inhibitory peptides from *Chouguiyu*

**Figure 1:**
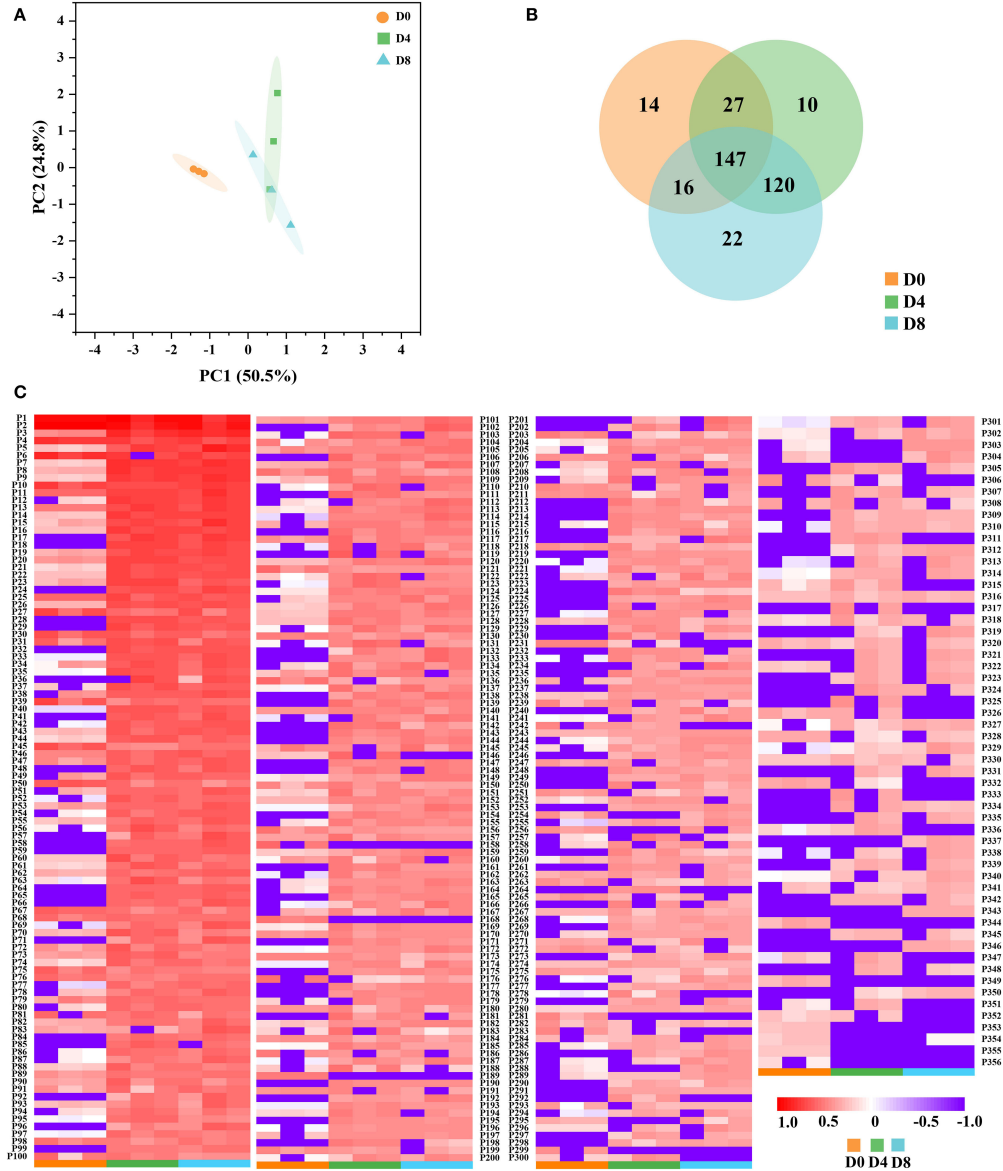
Identification of angiotensin I converting enzyme (ACE) inhibitory peptides from *Chouguiyu* at different fermentation times. **(A)** Principal component analysis (PCA), **(B)** Venn, and **(C)** heatmap analysis of ACE inhibitory peptides in the D0, D4, and D8 fermentation groups.

In this study, the ACE inhibition rates of the peptide extract from *Chouguiyu* at different fermentation times are shown in Supplementary Figure S1. Interestingly, the peptide extract in *Chouguiyu* exhibited good ACE inhibition activity as same as the positive drug captopril. The maximum ACE inhibition rate was observed in the D8 group, reaching 80.46%, significantly higher than that in the D0, D4, and positive control groups. To confirm which peptides contributed to the ACE inhibition activity, peptidomic analysis was used to identify the ACE inhibitory peptides. A previous study found that small-molecule peptides (generally ≤ 10 AAs) with various hydrophobic amino acids in the structure possessed good ACE inhibitory activity (33). A large number of ACE inhibitory peptides were found by virtual screening (8, 34). AHTpin was a common virtual method to predict and design efficient antihypertensive peptides (6, 35). In this study, a total of 356 potential ACE inhibitory peptides (AAs ≤ 10) were discovered by AHTpin. The principal component analysis (PCA) plot showed that the unfermented group (D0) was significantly different from the fermented groups (D4 and D8) (Figure 1A). Similar results were found in this study (Figure 1B), and more common ACE inhibitory peptides (120) were found in the groups D4 and D8. These bioactive peptides were named from P1 to P356 in order of their abundance (Figure 1B and Supplementary Table S1). Most of them increased with the increase of fermentation time, reaching their peaks in the D8 group. The most common ACE inhibitory peptides are rich in hydrophobic amino acids, and aromatic amino acids and aliphatic amino acids are conducive to enhancing ACE inhibitory activity (22, 36). In this study, plenty of hydrophobic amino acids were found in the ACE inhibitory peptides, of which 178 peptides possessed a proportion of hydrophobic amino acids in their sequences exceeding 50.00% (Supplementary Table S1). Valine, isoleucine, and proline were the main hydrophobic amino acids in the sequences of ACE inhibitory peptides (Supplementary Table S1).

### Precursor proteins and their cleavage locations of ACE inhibitory peptides from *Chouguiyu*

**Figure 2:**
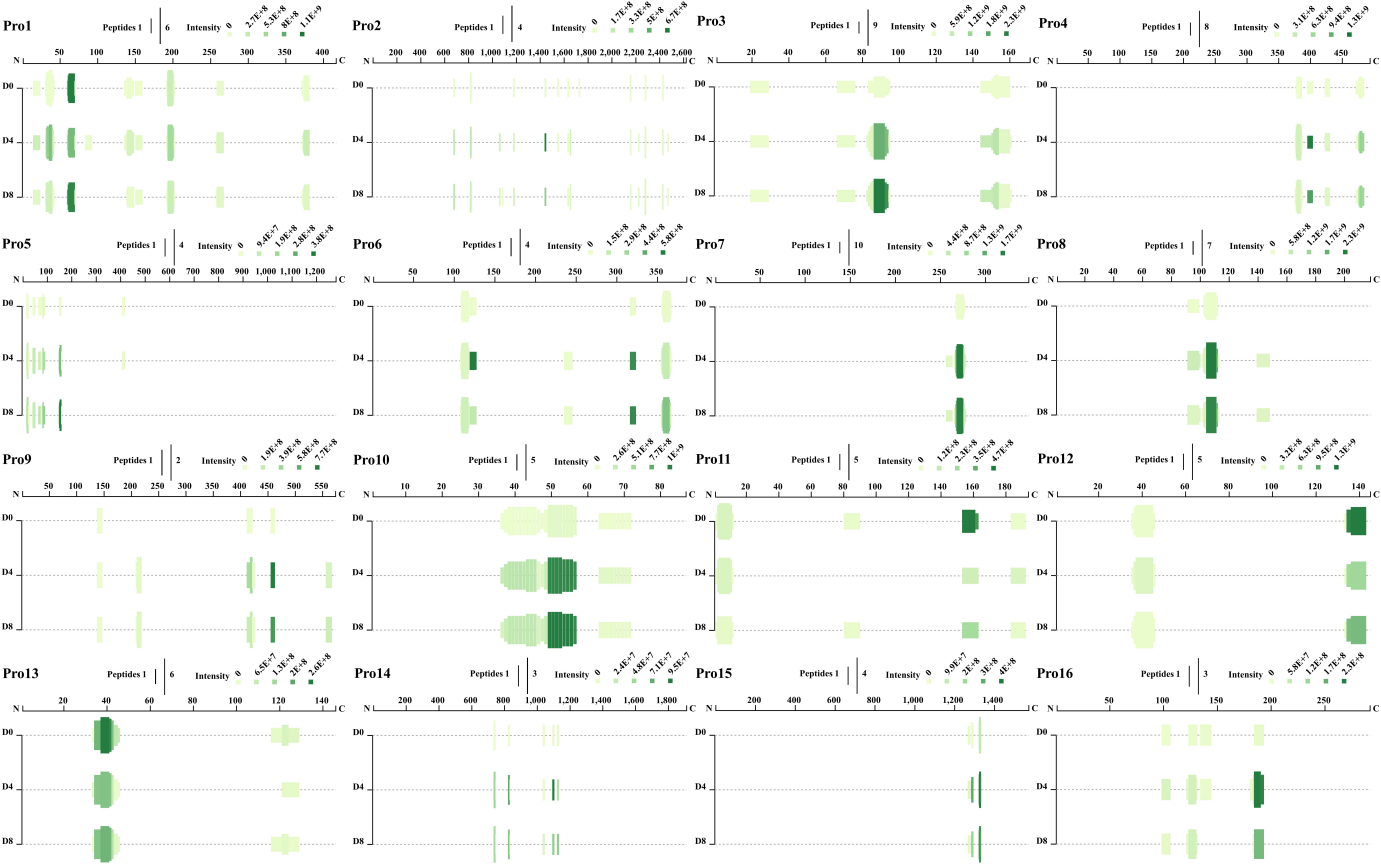
Angiotensin I converting enzyme inhibitory peptide profile of representative precursor proteins (Pro1–Pro16) during fermentation of *Chouguiyu*.

A total of 94 kinds of precursor proteins were identified after comparison with the proteins of *Siniperca chuatsi* (Supplementary Table S2). As shown in Figure 2, many ACE inhibitory peptides were derived from the same precursor protein at multiple locations, and most peptides shared the same cleavage locations with each other. The precursor proteins that could produce most ACE inhibitory peptides concentrated on muscle-type creatine kinase (27 peptides), nebulin (24 peptides), troponin I (16 peptides), myosin-binding protein H-like (14 peptides), fast skeletal muscle myosin heavy chain isoform 3 (14 peptides), nebulin isoform X8 (13 peptides), glyceraldehyde-3-phosphate dehydrogenase (11 peptides), troponin T (10 peptides), and nebulin (10 peptides), which were also important proteins of mandarin fish (Figure 2). The 10 peptides with high abundance including P1, P2, P3, P4, P5, P6, P7, P8, P9, and P10, derived from triosephosphate isomerase (location: 240–247), parvalbumin (location: 103–109), troponin I (location: 153–161), 14 kDa phosphohistidine phosphatase-like (location: 135–143), myosin heavy chain (location: 5–13), hemoglobin subunit alpha-B-like (location: 35–43), troponin T (location: 105–111), myosin-binding protein H-like (location: 424–430), nebulin isoform X8 (location: 364–371), and muscle-type creatine kinase (location: 60–69), respectively (Figure 2 and Supplementary Table S2).

### Characteristics of ACE inhibitory peptides from *Chouguiyu* by *in silico* analysis

The characteristics of thirty ACE inhibitory peptides with the highest abundance were further analyzed using *in silico* analysis, including isoelectric point, toxin, instability, hydrophobicity, water solubility, and aliphatic index (Supplementary Table S3). All the peptides were found to be novel peptides after searching from PepBank. All the ACE inhibitory peptides possessed non-toxicity, providing the possibility of safe use. A previous study showed that the hydrophobic peptides with small molecular weight from fish sauce had better ACE inhibition activity (12). In this study, the hydrophobicity of most ACE inhibitory peptides was more than 8.31 kcal/mol, indicating high hydrophobicity. At the same time, P1, P9, P25, P14, P20, P5, P24, P21, and P22 had high hydrophilicity index (>100), which indicated that they were highly amphiphilic and had the potential to be used in emulsion foods containing aqueous phase and lipid phase simultaneously (37). Most peptides had good water solubility and acidic isoelectric points. Similar studies found that many ACE inhibitory peptides exhibited acidic isoelectric points (37, 38). The stability of peptides plays a key role in maintaining their ACE inhibitory activities during digestion (39). The instability index ≥ 40 indicates that the peptides are considered to be unable to remain intact during digestion. In this study, a total of 18 peptides possessed low instability indexes (<40). According to the characteristics of ACE inhibitory peptides, P1, P2, P4, P7, P8, P9, and P10 were chosen as the core ACE inhibitory peptides for further analysis.

### Intermolecular interactions between ACE and core ACE inhibitory peptides from *Chouguiyu*

Molecular docking technology has been widely used to study the ACE inhibition mechanism of peptides (4, 8). In this study, the energy of molecular docking between ACE and ACE inhibitory peptides is shown in Supplementary Table S4. Lower docking energy (Δ*E*_docking_) indicates better affinity between the peptides and receptor (30, 40). In this study, the docking energy of seven ACE inhibitory peptides was less than −95 kcal/mol, far lower than that of positive control captopril (−15 kcal/mol) and the ACE inhibitory peptides in other reports (−3.86 kcal/mol) (13). The binding energy (Δ*E*_binding_) of peptides was all lower than −145 kcal/mol, suggesting their stable binding with ACE. The electrostatic energy (Δ*E*_ele_) of the peptides was much smaller than the van der Waals energy (Δ*E*_vdw_).

**Figure 3:**
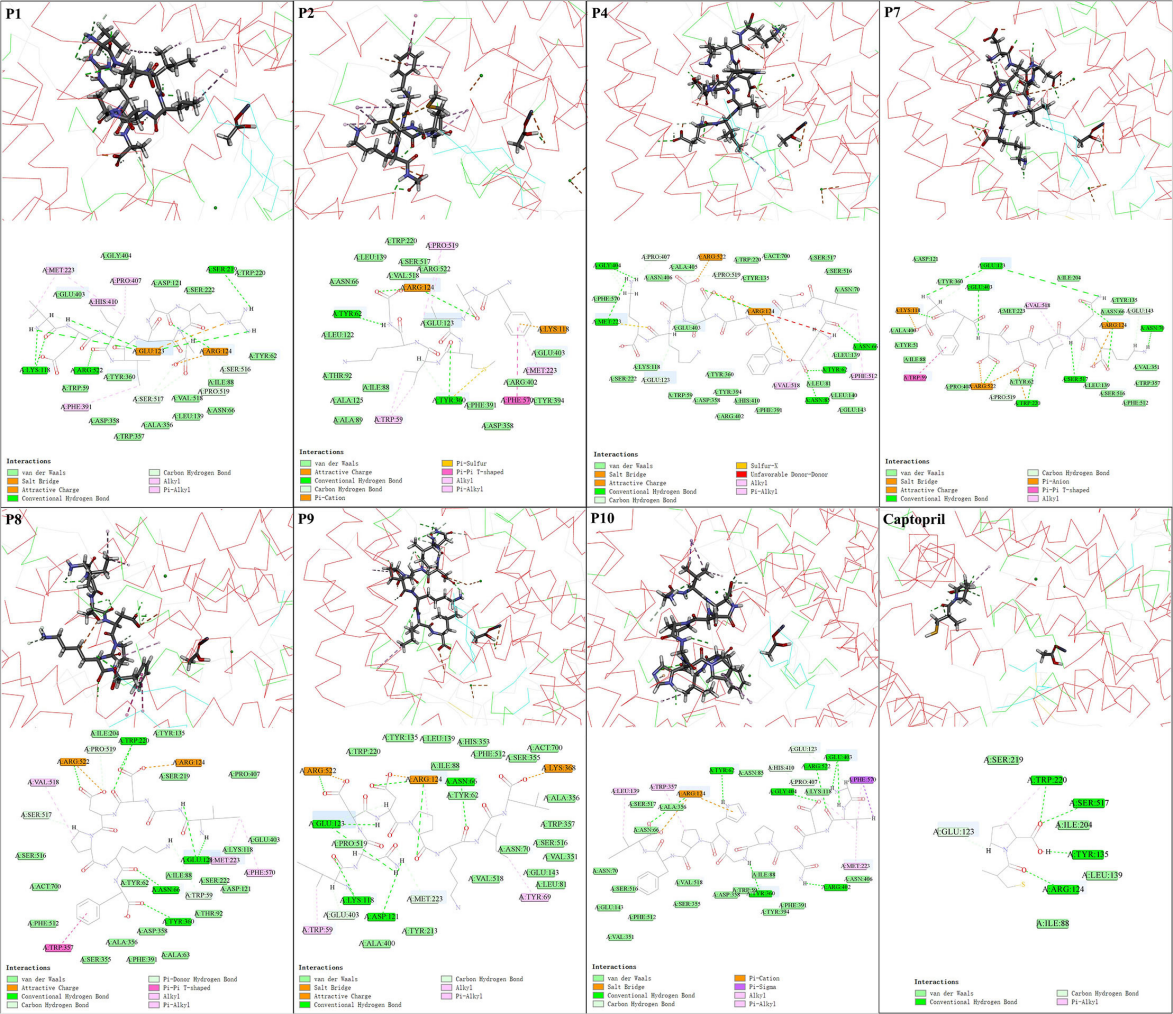
Molecular interactions of the core ACE inhibitory peptides (P1, P2, P4, P7, P8, P9, and P10) and captopril into the active site of ACE.

The inhibition mechanisms of ACE inhibitory peptides were explored by intermolecular interactions at the atomic level (Figure 3, Supplementary Figure S2, and Supplementary Table S5). More kinds of interactions were found in seven ACE inhibitory peptides than in captopril with ACE, suggesting their stronger interactions, in accordance with the results of molecular docking energy. There were 14 kinds of interactions found in complexes of seven ACE inhibitory peptides and ACE, belonging to electrostatic interaction (attractive charge, pi-anion, and pi-cation), hydrogen bond (salt bridge, conventional hydrogen bond, carbon-hydrogen bond, and pi-donor hydrogen bond), hydrophobic interaction (pi-pi T-shaped, pi-sigma, alkyl, and pi-alkyl), and other interaction (pi-sulfur and sulfur-X). Hydrogen bond and hydrophobic interaction are considered the main interactions between ACE inhibitory peptides and ACE (4, 8). In this study, plenty of salt bridges, conventional hydrogen, carbon-hydrogen bond, and alkyl were found between the 7 ACE inhibitory peptides and ACE. The residues in ACE for the formation of the salt bridge were concentrated on Arg522, Arg124, and Lys118, while the residues Tyr62, Asn66, Asn70, Lys118, Asp121, Glu123, Ser219, Trp220, Tyr360, Arg402, Glu403, Gly404, Ser517, and Arg522 contributed to forming a conventional hydrogen bond. Similar residues in ACE were reported in the formation of conventional hydrogen bonds with ACE inhibitory peptides (30). Among these residues, Arg522 played an important role in the ACE activity because it acted as the chloride ion ligand for ACE activation for substrate hydrolysis (40). The conventional hydrogen bond forming between these residues and peptides might lead to the ACE inactivation (40). In this study, the interaction between the ACE inhibitory peptides and ACE was further analyzed at the atomic level. The unsaturated oxygen atoms in P1 (O17, O51, O94, and O127), P2 (O31, O64, O108, and O109), P4 (O13, O14, O16, O93, O94, O106, and O129), P7 (O11, O13, O115, O42, O43, O54, O55, O88, and O114), P8 (O36, O37, O39, O48, O49, O86, and O106), P9 (O20, O43, O44, O55, O56, O72, O93, and O132), and P10 (O24, O34, O141, and O142) as H-acceptors formed strong interactions with ACE, contributing to the formation of the salt bridge and conventional hydrogen bond. Residues Trp59, Glu123, Glu143, Met223, Glu403, Pro407, His410, Ser516, Ser517, and Pro519 in ACE played a key role in forming a carbon-hydrogen bond with ACE inhibitory peptides. Furthermore, plenty of alkyl interactions were found in P1 (C8-Met223, C39-Met223, and C39-Pro407), P2 (C38-Pro519 and C42-Pro519), P4 (C30-Val518), P7 (C64-Val518), P8 (P8-Val518 and C15-Met223), P9 (P9-Arg124), and P10 (C15-Met223, C15-Pro407, and C129-Leu139). There were also some pi-alkyl interactions in P1 (C65-Phe391 and C46-His410), P2 (C71-Trp59, C75-Trp59, and P2-Met223), P4 (C30-Phe512), P8 (C15-Phe570), P9 (C8-Trp59), and P10 (P10-Trp357 and C15-Phe570). In addition, there was an unfavorable bond found in P4 (H2-Arg124), suggesting the formation of repulsive charge interaction.

### Correlation between core ACE inhibitory peptides and their cleavage proteases from microorganisms in *Chouguiyu*

**Figure 4:**
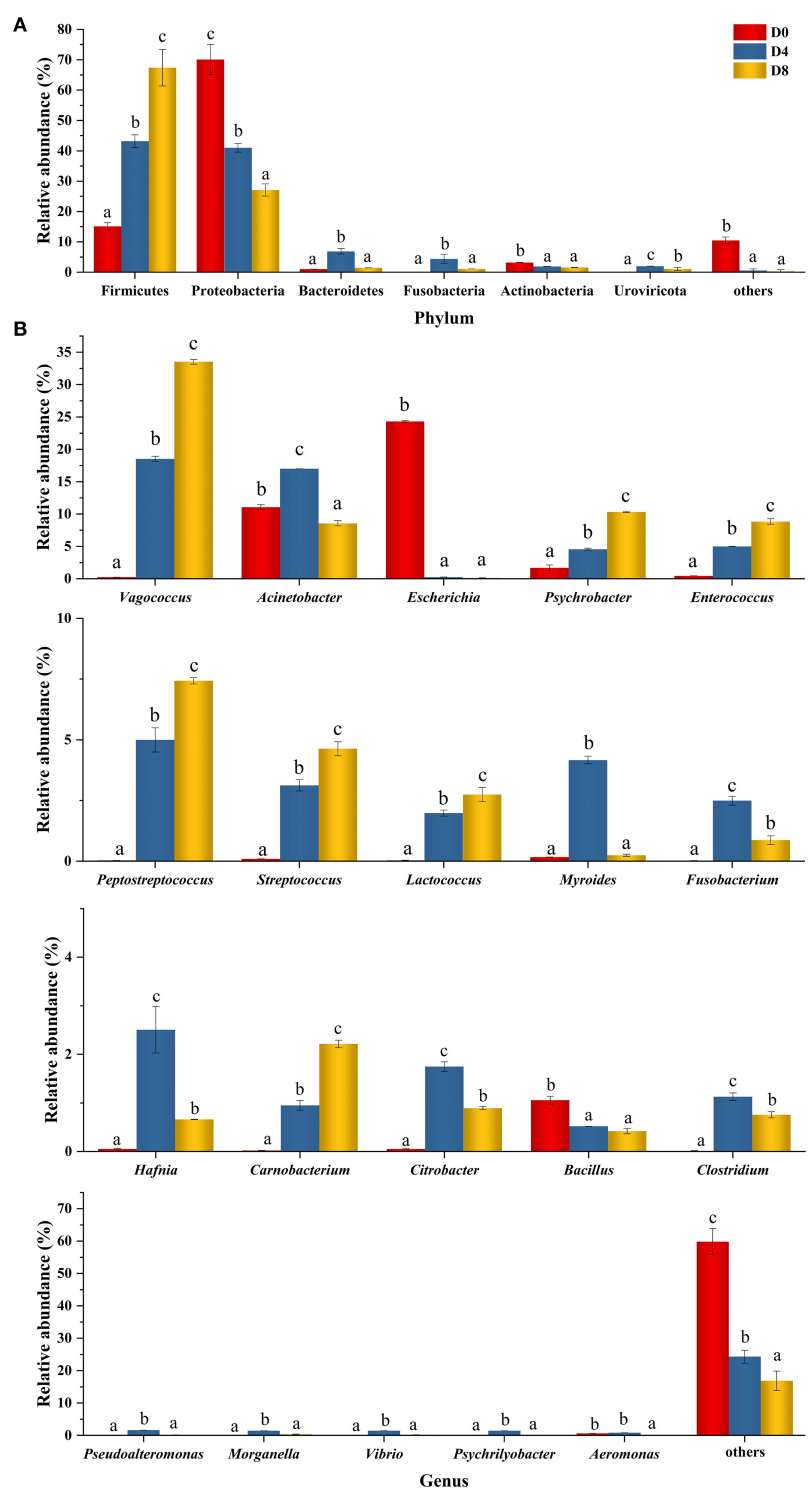
Changes in the relative abundance of the microbial community at the **(A)** phylum and **(B)** genus level in *Chouguiyu* during the fermentation process.

Peptides from aquatic products exhibit different structures and inhibition functions to concurrently inhibit multiple enzymes involved in chronic diseases (40). As an aquatic product, *Chouguiyu* is produced under the metabolism of the complex microbial community. In this study, the ACE inhibitory peptides with high activity might be formed by the hydrolysis of proteases from the microbial community. The relative abundance of the microbial community in *Chouguiyu* during the fermentation process was first studied (Figure 4). Six bacterial phyla were identified according to the relative abundance of more than 0.1%, including Firmicutes, Proteobacteria, Bacteroidetes, Fusobacteria, Actinobacteria, and Uroviricota (Figure 4A). Among these, Firmicutes and Proteobacteria were the dominant phyla with a total proportion of over 84% in the whole fermentation process. Proteobacteria possessed the highest relative abundance at the beginning of fermentation (D0) reaching 70.09% and declined with the increasing fermentation time, while Firmicutes gradually occupied the main abundance and increased to 67.37% at the end of fermentation (D8). Similar dominant phyla were reported in other studies on the *Chouguiyu* (41). A total of 438 microbial genera with a relative abundance of more than 0.1% were found in *Chouguiyu* in the D0, D4, and D8 groups. The 20 genera with the highest total relative abundance of the D0, D4, and D8 groups are shown in Figure 4B. Few dominant bacteria were observed in the group D0, only including *Escherichia* (24.40%), *Acinetobacter* (11.09%), *Psychrobacter* (1.71%), and *Bacillus* (1.06%), while the relative abundance of other genera was all less than 1%. With the increasing fermentation time, the abundance of *Escherichia* and *Bacillus* significantly decreased, and more microbial genera with high abundance occurred. In the D4 group, there were 16 genera with an abundance of more than 1%, mainly including *Vagococcus* (18.79%), *Acinetobacter* (17.23%), *Enterococcus* (5.06%), *Peptostreptococcus* (5.06%), and *Psychrobacter* (4.63%). After fermentation for 8 days, the abundance of *Vagococcus, Psychrobacter*, and *Enterococcus* reached the maximum, respectively, reaching 33.77, 10.40, and 8.91%. Besides, the genera with an abundance of more than 1% in the D8 group also included *Acinetobacter* (8.63%), *Peptostreptococcus* (7.48%), *Streptococcus* (4.66%), *Lactococcus* (2.76%), and *Carnobacterium* (2.23%). Similar dominant genera such as *Vagococcus, Psychrobacter, Lactococcus, Fusobacterium, Vibrio*, and *Psychrilyobacter* were found in the late fermentation period in *Chouguiyu* (41).

**Figure 5:**
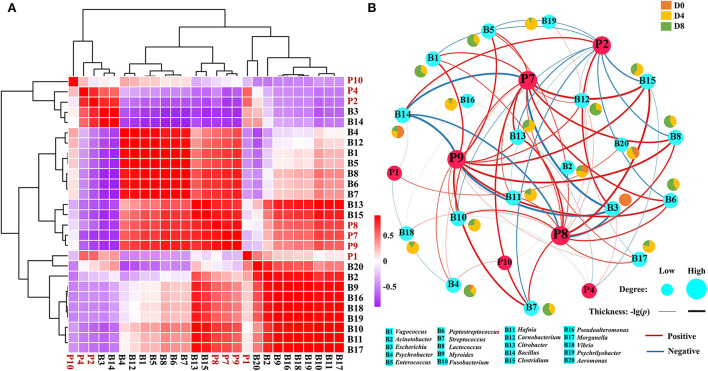
Correlation analysis between the ACE inhibitory peptides and microbial genera. **(A)** Clustering heatmap and **(B)** correlation network (|*r*| > 0.6 and *p* < 0.05) based on Pearson's correlation tests.

To explore the possible formation mechanism of core ACE inhibitory peptides, the correlation between 20 microbial genera and 7 core ACE inhibitory peptides was then analyzed by Pearson's correlation (Figure 5A). For microbial genera, three distinct clusters were observed, including microbial cluster 1 (*Escherichia* and *Bacillus*), microbial cluster 2 (*Psychrobacter, Carnobacterium, Vagococcus, Enterococcus, Lactococcus, Peptostreptococcus, Streptococcus, Citrobacter*, and *Clostridium*), and microbial cluster 3 (*Aeromonas, Acinetobacter, Myroides, Pseudoalteromonas, Vibrio, Psychrilyobacter, Fusobacterium, Hafnia*, and *Morganella*). Interestingly, most ACE inhibitory peptides in *Chouguiyu* showed negative relations with microbial cluster 1 but positive correlations with the genera in microbial cluster 2 and microbial cluster 3. The correlation network map was then built using the Pearson's correlation coefficient between the ACE inhibitory peptides and microbial genera (|*r*|>0.6 and *p* < 0.05) (Figure 5B). Meanwhile, the topological characteristics of the correlation network map were analyzed using R, including the betweenness centrality, closeness centrality, degree, neighborhood connectivity, topological coefficient, and average shortest path length (Supplementary Figure S3). Among these, the degree is the main metric to describe the centrality of nodes in the correlation network, and a higher degree indicates more importance in the network (42). Interestingly, P8, P7, P9, and P2 possessed the highest degree and were significantly positively correlated with the most microbial genera (16, 15, 14, and 13, respectively), while P1, P4, and P10 showed a significantly positive relation with less microbial genera, mainly including *Escherichia* and *Bacillus*.

**Figure 6:**
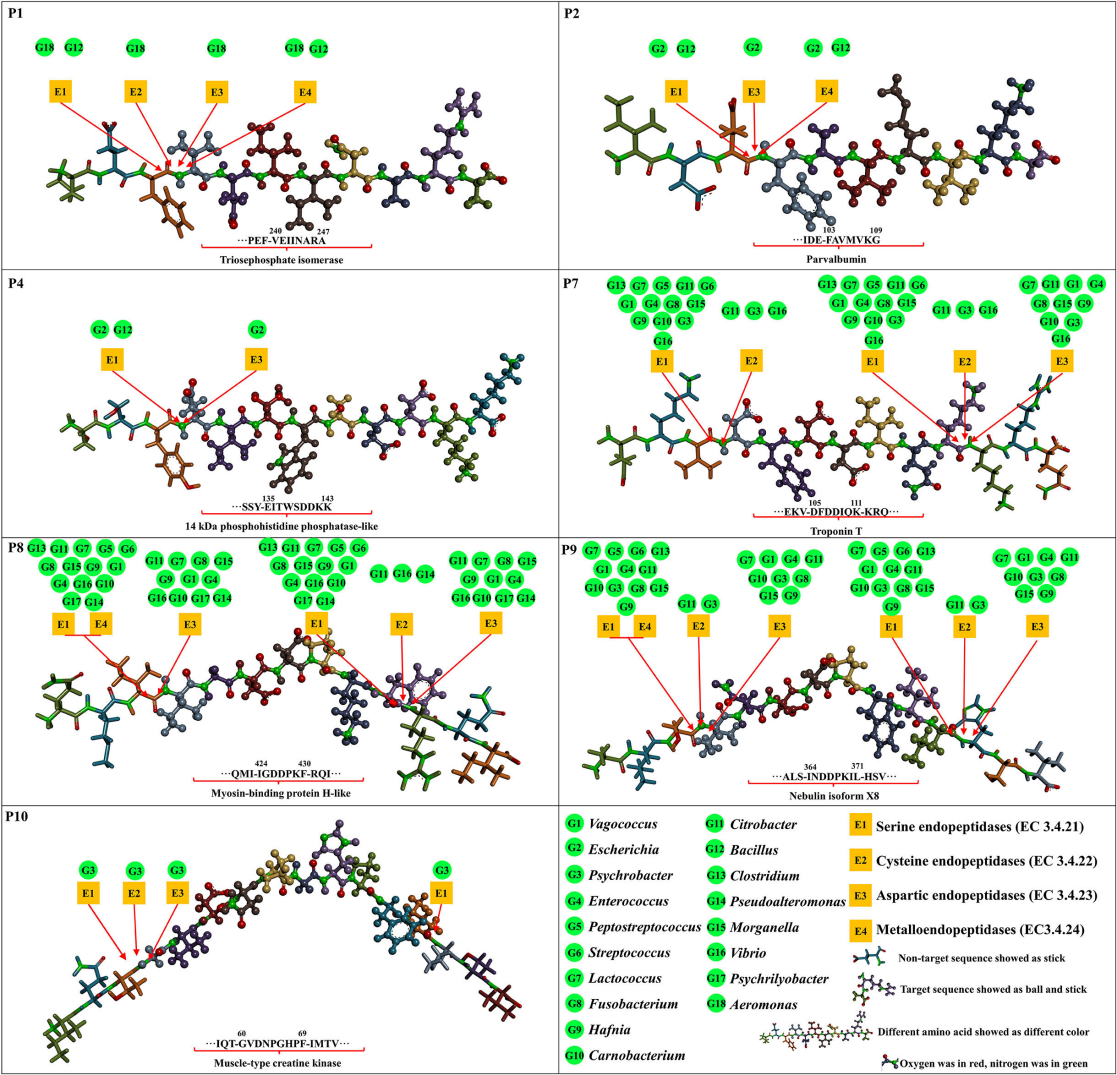
Formation of the core ACE inhibitory peptides (P1, P2, P4, P7, P8, P9, and P10) through cleavage proteases from the microbial genera based on hydrolysis prediction and correlation network.

After prediction at BIOPEP-UWM, the cleavage proteases from the microbial genera in *Chouguiyu* for preparing 7 core ACE inhibitory peptides were further analyzed (Figure 6 and Supplementary Table S6). P1 could be produced by serine endopeptidases (COG1770, COG0466, COG1219, COG0265, and COG0681), cysteine endopeptidases (COG3672), aspartic endopeptidases (COG0616 and COG3577), and metalloendopeptidases (COG0826, COG0750, COG0501, COG4942, and COG1164) from *Aeromonas* (*r* = 0.69), as well as by serine endopeptidases (COG1506, COG0542, COG1404, COG1505, and ENOG410XP7P) and metalloendopeptidases (COG5504, COG3340, COG0465, COG3740, and COG0501) from *Bacillus* (*r* = 0.62). P2 could be prepared by serine endopeptidases (COG1770, ENOG410XP7P, COG0542, COG0265, and ENOG4111MMN), aspartic endopeptidases (COG0616), and metalloendopeptidases (COG0739, ENOG410XZ09, COG0465, and COG0339) from *Escherichia* (*r* = 0.84) and by serine endopeptidases (COG1506, COG0542, COG1404, COG1505, and ENOG410XP7P) and metalloendopeptidases (COG5504, COG3340, COG0465, COG3740, and COG0501) from *Bacillus* (*r* = 0.82). P4 could be obtained by serine endopeptidases (COG1770, NOG410XP7P, COG0542, COG0265, and ENOG4111MMN) and aspartic endopeptidases (COG0616) from *Escherichia* (*r* = 0.63), as well as by serine endopeptidases (COG1506, COG0542, COG1404, COG1505, and ENOG410XP7P) from *Bacillus* (*r* = 0.60). Many microbial genera could secrete various proteases for P7, P8, and P9 production. These proteases were concentrated on serine endopeptidases from *Clostridium, Lactococcus, Peptostreptococcus, Citrobacter, Streptococcus, Vagococcus, Enterococcus, Fusobacterium, Morganella, Hafnia, Carnobacterium*, and *Psychrobacter* and aspartic endopeptidases from *Lactococcus, Citrobacter, Vagococcus, Enterococcus, Fusobacterium, Morganella, Hafnia, Carnobacterium*, and *Psychrobacter*. In addition, fewer microbial genera could produce cysteine endopeptidases for P7, P8, and P9 production, mainly including *Citrobacter* (COG3672) and *Psychrobacter* (ENOG4111IPD). For P10, only *Psychrobacter* (*r* = 0.64) contributed most to the generation of P10 by serine endopeptidases (COG0466, COG0542, COG1219, COG1067, and COG0265), cysteine endopeptidases (ENOG4111IPD), and aspartic endopeptidases (COG0616).

### Formation and inhibition mechanism of core ACE inhibitory peptides from *Chouguiyu*

In this study, the peptide extract from *Chouguiyu* exhibited good ACE inhibition, and the inhibition rate was significantly enhanced after fermentation for 8 days (Supplementary Figure S1). To identify the ACE inhibitory peptides in *Chouguiyu*, peptidomics and AHTpin were combined, and 356 ACE inhibitory peptides were obtained (Figure 1). P1, P2, P4, P7, P8, P9, and P10 were selected as the core ACE inhibitory peptides according to their abundance, characteristics, and docking energy. Their precursor proteins were triosephosphate isomerase, parvalbumin, 14 kDa phosphohistidine phosphatase-like, troponin T, myosin-binding protein H-like, nebulin isoform X8, and muscle-type creatine kinase, respectively (Figure 2 and Supplementary Table S2). Based on the hydrolysis prediction, these 7 core peptides could be hydrolyzed from various proteases derived from plenty of microorganisms (Supplementary Table S6). Lactic acid bacteria (LAB) were considered an important group of microorganisms in *Chouguiyu*, contributing greatly to its aroma formation (43). After the further correlation analysis between these proteases and ACE inhibitory peptides, the P7, P8, and P9 were mainly produced by the proteases from LAB including *Lactococcus, Enterococcus, Vagococcus, Peptostreptococcus*, and *Streptococcus*. These genera were also reported for the formation of umami peptides in *Chouguiyu* (26). Differently, the P1, P2, P4, and P10 were mainly produced by *Aeromonas, Bacillus, Escherichia*, and *Psychrobacter*. *Bacillus* is famous for its ability to produce various high-activity proteases (44). The high abundance of these genera at the beginning of fermentation time was helpful in producing these four ACE inhibitory peptides. The high abundance of *Vagococcus, Lactococcus, Fusobacterium, Streptococcus, Escherichia, Bacillus*, and *Pseudoalteromonas* contributed to the production of the core ACE inhibitory peptides. The high abundance of these genera was also reported in *Chouguiyu* in other studies (41, 45, 46). The isolation of specific microorganisms is expected to directionally produce the responding ACE inhibitory peptides.

Intermolecular interaction analysis by molecular docking is considered an effective technology to reveal the inhibition mechanism of ACE inhibitory peptides on ACE (8). In this study, molecular docking between ACE inhibitory peptides and ACE showed that the amino acid residues at the end of peptides played an important role in their ACE inhibitory activity. Previous studies showed that the amino acid residues at N-terminal in ACE inhibitory peptides could form strong interactions with ACE (22, 47). Similar results were also observed in this study that V- in P1 formed 3 conventional hydrogen bonds, 1 carbon-hydrogen bond, and 1 alkyl; E- in P4 formed 1 salt bridge and 1 conventional hydrogen bond; D- in P7 formed 1 salt bridge, 2 conventional hydrogen bond, and 1 carbon-hydrogen bond; I- in P8 formed 1 conventional hydrogen bond and 1 alkyl; I- in P9 formed 1 conventional hydrogen bond, 2 carbon-hydrogen bond, and 1 pi-alkyl; G- in P10 formed 1 conventional hydrogen bond and 1 carbon-hydrogen bond (Figure 3 and Supplementary Table S5). Meanwhile, the positively charged (13, 48) and hydrophobic (49) amino acid residues at C-terminal were beneficial to their ACE inhibitory activity. Similar structures were observed in this study, including the positively charged residues (-A in P1, -K in P4, and -K in P7) and hydrophobic residues (-F in P8, -L in P9, and -I in P10). These residues could perform a good ACE inhibitory activity, probably due to the formation of plenty of salt bridges, conventional hydrogen bonds, carbon-hydrogen bonds, and alkyl with ACE (Figure 3 and Supplementary Table S5). The illustration of the formation and inhibition mechanism of ACE inhibitory peptides in *Chouguiyu* provides important technical support for the isolation of the proteases and microbial strains to directionally produce the responding ACE inhibitory peptides and new ideas to screen novel ACE inhibitory peptides from other fermented foods.

## Conclusion

Peptide extract of *Chouguiyu* exhibited a good inhibition effect on ACE, especially in the D8 group. A total of 356 ACE inhibitory peptides were predicted using *in silico*, and most ACE inhibitory peptides increased with the increase of fermentation time. These peptides could be obtained from 94 kinds of precursor proteins which were concentrated on muscle-type creatine kinase, nebulin, and troponin I. Seven core ACE inhibitory peptides with high abundance, stable structure, high hydrophobicity, and low docking energy were selected, including P1 (VEIINARA), P2 (FAVMVKG), P4 (EITWSDDKK), P7 (DFDDIQK), P8 (IGDDPKF), P9 (INDDPKIL), and P10 (GVDNPGHPFI). Molecular docking showed that the inhibition of ACE mainly resulted from unsaturated oxygen atoms in the seven peptides forming a salt bridge and conventional hydrogen bond with ACE as H-acceptors, followed by active carbon atoms forming alkyl and pi-alkyl interactions. The N-terminal and C-terminal amino acid residues of these peptides played an important role in their ACE inhibitory activity. P7, P8, and P9 were mainly produced by the proteases from LAB including *Lactococcus, Enterococcus, Vagococcus, Peptostreptococcus*, and *Streptococcus*, while P1, P2, P4, and P10 were mainly produced by *Aeromonas, Bacillus, Escherichia*, and *Psychrobacter* after analysis by hydrolysis prediction and correlation network. This study provides a theoretical basis for understanding the formation and inhibition mechanism of ACE inhibitory peptides in *Chouguiyu* and important information for the related microbial strains and proteases to produce the responding ACE inhibitory peptides.

## Data availability statement

The data presented in the study are deposited in ProteomeXchange, accession number PXD033251.

## Ethics statement

Ethical review and approval was not required for the animal study because Frozen mandarin fish were used in this study.

## Author contributions

DY: methodology and writing – original draft. LL: conceptualization. CL: writing – review and editing and conceptualization. SC and JD: visualization. SY: data curation. All authors contributed to the article and approved the submitted version.

## Funding

This research was financially supported by the National Key R&D Program of China (2019YFD0901903), the China Agriculture Research System of MOF and MARA (CARS-46), the Guangdong Basic and Applied Basic Research Foundation (2021A1515010872), the Young S&T Talent Training Program of Guangdong Provincial Association for S&T, China (SKXRC202210), and the Central Public-interest Scientific Institution Basal Research Fund, CAFS (2020TD69).

## Conflict of interest

The authors declare that they have no known competing financial interests or personal relationships that could have appeared to influence the work reported in this study.

## Publisher's note

All claims expressed in this article are solely those of the authors and do not necessarily represent those of their affiliated organizations, or those of the publisher, the editors and the reviewers. Any product that may be evaluated in this article, or claim that may be made by its manufacturer, is not guaranteed or endorsed by the publisher.
