# Supplementary material for: Formation and inhibition mechanism of novel angiotensin I converting enzyme inhibitory peptides from Chouguiyu
Source: Front Nutr. 2022 Jul 22;9:920945. doi: 10.3389/fnut.2022.920945 (PMC9355153; doi:10.3389/fnut.2022.920945)
Supplement: Supplementary file 1 [file Data_Sheet_1.PDF]

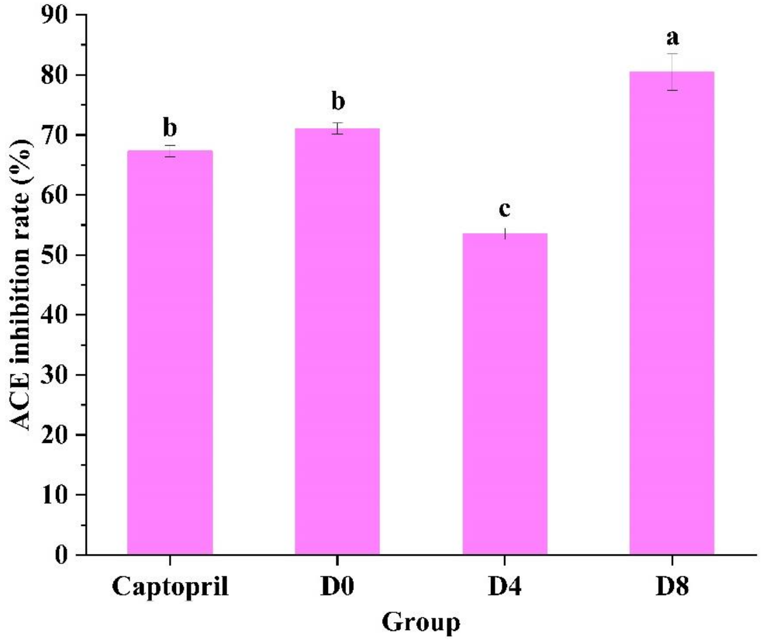


**Figure S1.** ACE inhibition rate of peptide extract in *Chouguiyu* at different fermentation time. Bars labelled with different letters are statistically different ($p<0.05$), as tested by one-way ANOVA and the Tukey test.
