# Supplementary figures and images for: Formation and inhibition mechanism of novel angiotensin I converting enzyme inhibitory peptides from Chouguiyu
Source: Front Nutr. 2022 Jul 22;9:920945. doi: 10.3389/fnut.2022.920945 (PMC9355153; doi:10.3389/fnut.2022.920945)

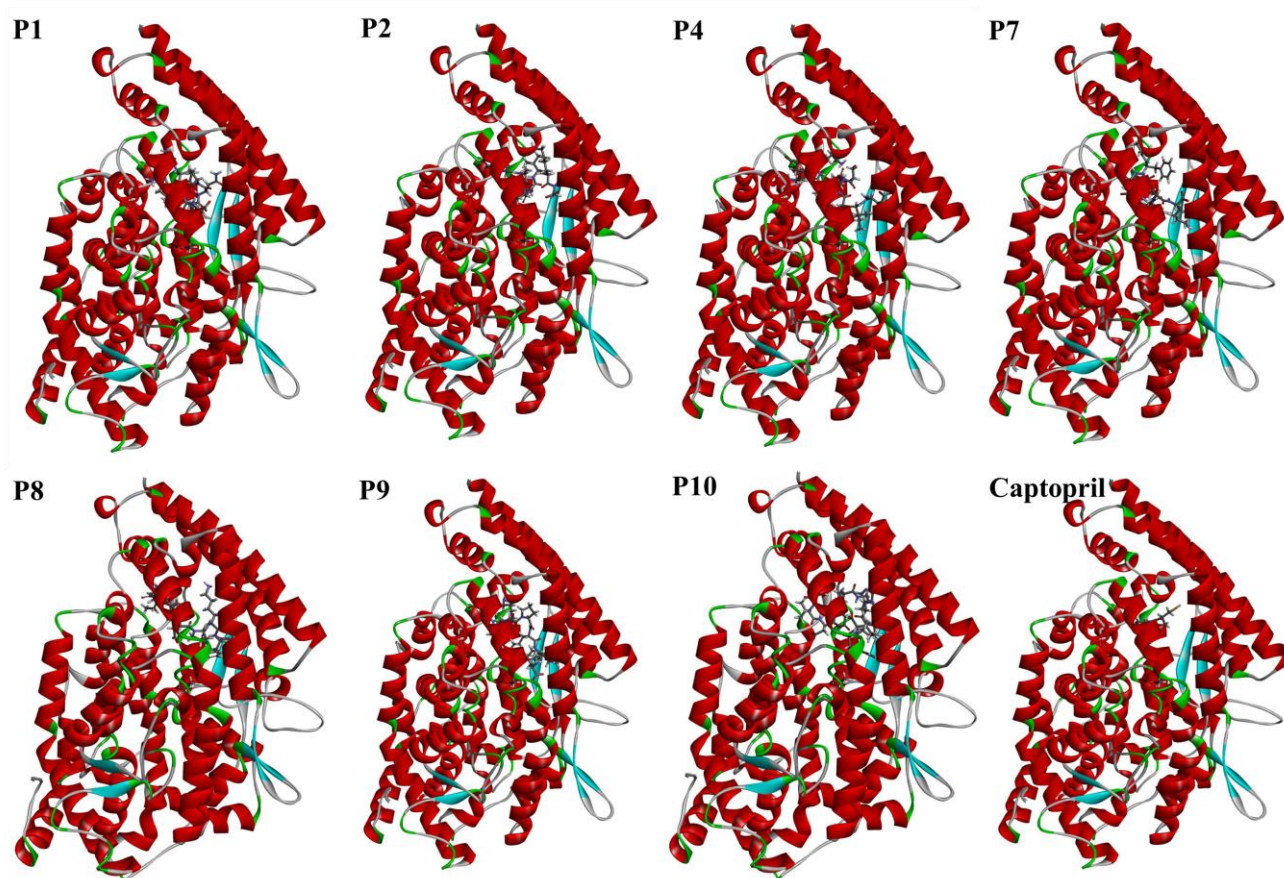

**Figure S2.** 3D complex of molecular docking between ACE and ACE inhibitory peptides or captopril.

Supplement: Supplementary file 2 [file Data_Sheet_2.PDF]

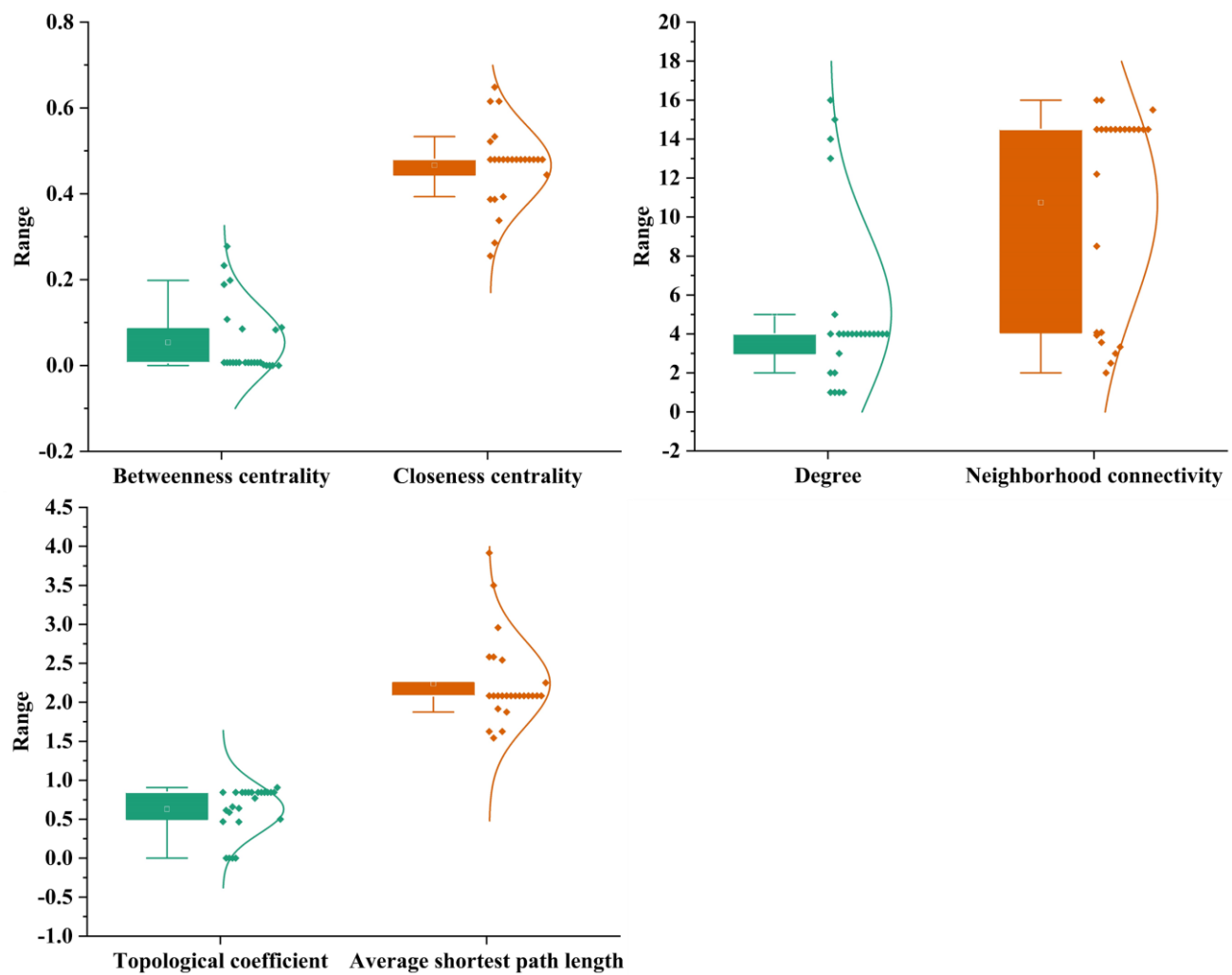

**Figure S3.** The topological features of the correlation network map

Supplement: Supplementary file 3 [file Data_Sheet_3.PDF]
