# Supplementary material for: Formation and inhibition mechanism of novel angiotensin I converting enzyme inhibitory peptides from Chouguiyu
Source: Front Nutr. 2022 Jul 22;9:920945. doi: 10.3389/fnut.2022.920945 (PMC9355153; doi:10.3389/fnut.2022.920945)
Supplement: Supplementary file 4 [file Data_Sheet_4.PDF]

Table S1 Sequence, SVM score and molecular weight of ACE inhibitory peptides from *Chouguiyu*

Peptide number	Peptide sequence	SVM score	Proportion of hydrophobic AAs	Molecular weight (Da)
P1	VEIINARA	0.04	62.50	885.52
P2	FAVMVKG	0.27	71.43	751.42
P3	DWRKNIEDK	0.68	22.22	1203.61
P4	EITWSDDKK	0.45	22.22	1121.55
P5	AAIYLRKPE	0.49	66.67	1060.61
P6	VAYPQTKTY	0.29	55.56	1070.55
P7	DFDDIQK	0.19	28.57	880.40
P8	IGDDPKF	0.44	42.86	791.39
P9	INDDPKIL	0.24	50.00	927.51
P10	GVDNPGHPFI	0.01	50.00	1052.52
P11	EEVGVEEEKP	0.32	30.00	1144.54
P12	VGVEEEKP	1.02	37.50	886.45
P13	FPSIVGRP	1.11	62.50	872.50
P14	QDDPLLVH	0.22	50.00	936.48
P15	MDALKK	0.10	50.00	747.41
P16	GDWRKNIEDK	0.17	20.00	1260.63
P17	EKVDFDDIQK	0.78	30.00	1236.61
P18	VGVEEEKPKF	0.12	40.00	1161.62
P19	DDMEKIWHHT	0.18	30.00	1311.58
P20	LPHDTPL	0.64	57.14	792.43
P21	YDDIKKVVKA	2.77	50.00	1178.68
P22	AKYDDIKKVV	2.77	50.00	1178.68
P23	DMEKIWHHT	0.42	33.33	1196.55
P24	KYDDIKKVV	2.54	44.44	1107.64
P25	IPVISVPEKK	1.96	60.00	1109.69
P26	DFTEPPK	0.45	42.86	833.40
P27	YKATEPVIAF	0.18	70.00	1138.61
P28	KPSAPKIP	0.11	62.50	837.52
P29	EPEGIKSPP	1.12	44.44	953.49
P30	VDNPGHPFI	0.51	55.56	995.49
P31	FDKPVSPL	1.13	62.50	902.50
P32	YDDIKKVV	2.40	50.00	979.55
P33	EHDFTEPPK	0.59	33.33	1099.51
P34	VDFDDIQK	0.56	37.50	979.47
P35	EVGVEEEKP	0.63	33.33	1015.49
P36	IGEDPKY	1.36	42.86	821.40
P37	DDPLLVH	0.10	57.14	808.42
P38	DIDIRKDLY	0.12	44.44	1150.61
P39	IVYPWTQRH	0.72	55.56	1199.63
P40	EIPTKVPKAE	0.67	50.00	1111.64

P41	KVDFDDIQK	1.20	33.33	1107.57
P42	IGEDPKYL	0.55	50.00	934.49
P43	INDDPKILH	0.35	44.44	1064.57
P44	KAGFAGDDAP	0.34	50.00	948.44
P45	VAYPQTKT	0.14	50.00	907.49
P46	DPFRGGHY	0.76	37.50	948.43
P47	YLRKPE	0.74	50.00	805.46
P48	KPSAPKIPD	0.23	55.56	952.55
P49	LPVKDPVVD	0.77	66.67	981.56
P50	KEAARQIDGP	0.60	40.00	1084.57
P51	HESVDVIKVK	0.80	40.00	1153.66
P52	NDDPKIL	0.44	42.86	814.43
P53	DIRKDLY	0.26	42.86	922.50
P54	AVYLRKPE	0.20	62.50	975.56
P55	AVFPSIVGRP	0.54	70.00	1042.60
P56	KVLTKEIYA	0.08	55.56	1064.64
P57	AVGKVIPELN	0.25	60.00	1039.61
P58	KYDDIKKVVK	2.23	40.00	1235.74
P59	DKEIEDLKIK	0.79	30.00	1230.69
P60	DSMIPAQK	0.58	50.00	889.44
P61	KVEEEFPDL	0.07	44.44	1105.54
P62	YDDIKKVVK	2.54	44.44	1107.64
P63	KVLTKEIY	0.27	50.00	993.60
P64	APPHIFS	0.83	71.43	768.40
P65	NPYKEIDVS	1.08	44.44	1064.53
P66	LQDDPLLVH	0.30	55.56	1049.56
P67	VDNPGHPFIM	0.15	60.00	1126.53
P68	TVFPQTKT	0.24	37.50	921.50
P69	VGKVIPE	0.85	57.14	741.45
P70	GEKMTEPEID	0.52	30.00	1148.51
P71	VGKVIPELN	0.44	55.56	968.58
P72	IPVVDDK	1.00	57.14	785.44
P73	DANLKPIKPM	0.38	60.00	1126.63
P74	DVAGNVDYK	0.04	44.44	980.47
P75	LIVYPWTQRH	0.35	60.00	1312.72
P76	TVFPQTKTY	0.39	44.44	1084.57
P77	VYLRKPE	0.53	57.14	904.53
P78	YDEAGPSIVH	0.29	50.00	1087.51
P79	KIEEIFKKH	0.17	33.33	1171.68
P80	EEDIHPRNPP	1.63	40.00	1203.58
P81	LFDKPVSPL	1.00	66.67	1015.58
P82	IPheSVDVIK	0.84	50.00	1136.63
P83	YNELRVAP	0.62	62.50	961.51
P84	PEGTGTIKKQ	0.64	20.00	1058.58
P85	SLPHDTPL	0.07	50.00	879.46
P86	IPPEKPIKIP	1.28	70.00	1131.71

P87	ADKEIEDLK	0.61	33.33	1060.55
P88	SEYIPKPIKK	1.21	50.00	1202.71
P89	IPVNVDK	0.95	57.14	784.46
P90	APVEVVKP	1.76	75.00	838.50
P91	TPVRPVSP	0.86	62.50	852.49
P92	GKVIPELN	0.25	50.00	869.51
P93	AGFAGDDAPR	0.24	50.00	976.45
P94	VFPSIVGRP	0.83	66.67	971.57
P95	KEHDFTEPP	0.59	33.33	1099.51
P96	AIYLRKPE	0.73	62.50	989.58
P97	EVKQPAVADA	0.48	60.00	1027.54
P98	LRKPEKERIE	0.66	30.00	1297.76
P99	IGSLDVEKAK	0.07	40.00	1059.60
P100	LFDKPVSP	1.13	62.50	902.50
P101	EAGPSIVH	0.43	50.00	809.42
P102	IYLRKPE	0.67	57.14	918.54
P103	GFTLPPHNSR	0.41	40.00	1125.58
P104	IKEASGPINF	0.32	50.00	1075.58
P105	SKQEYDEAGP	0.54	30.00	1123.49
P106	SQLKEVVKLK	0.65	40.00	1171.74
P107	FPSIVGR	0.02	57.14	775.45
P108	MVQDVLK	0.07	57.14	832.46
P109	DWRKNIEDKA	0.98	30.00	1274.65
P110	VGMGQKDSY	0.08	33.33	984.45
P111	GEKMTEPEI	0.43	33.33	1033.49
P112	VGKVIPEL	0.38	62.50	854.53
P113	HDFTEPPKFT	0.29	40.00	1218.58
P114	VKVGNEMVTK	0.28	40.00	1104.61
P115	EVFKDLLDP	0.68	55.56	1075.57
P116	NPYKEIDV	1.63	50.00	977.49
P117	IKVPESDDL	0.96	44.44	1015.53
P118	MEIKHFKP	0.64	50.00	1029.56
P119	LPKSLEDDP	0.99	44.44	1013.51
P120	EVTLPKVDF	0.39	55.56	1047.57
P121	VVSALGKQYH	0.21	50.00	1101.61
P122	KVLTKEIYAK	0.45	50.00	1192.73
P123	EPAEAVGDWR	0.23	50.00	1129.53
P124	DDIKKVVKAA	2.03	50.00	1086.65
P125	IQSDNWYKEA	0.61	40.00	1253.58
P126	DDMEKIWHH	0.52	33.33	1210.53
P127	APKIPDGE	1.49	50.00	826.43
P128	DNPGHPFI	0.34	50.00	896.43
P129	EDDIHPRNPP	1.07	40.00	1189.56
P130	TWSDDKK	0.22	14.29	879.42
P131	KEIYAKL	0.26	57.14	864.52
P132	DKEIEDLKI	0.62	33.33	1102.60
P133	AVGKVIPEL	0.36	66.67	925.57

P134	FPEYDGK	0.19	42.86	855.39
P135	NQVSKAIDA	0.07	44.44	945.50
P136	PQTKTYFSH	0.08	33.33	1108.54
P137	TAIPMIPHQ	1.04	66.67	1007.53
P138	EDIAKGYDMK	0.99	40.00	1169.55
P139	APVEVVKPA	1.59	77.78	909.54
P140	FGGRNPFGQF	0.51	40.00	1126.54
P141	GFAGDDAPR	0.26	44.44	905.41
P142	EKMTEPEID	0.38	33.33	1091.49
P143	EVPEVYR	0.89	57.14	891.46
P144	TMKIQPLPQ	0.38	55.56	1055.59
P145	MKIQPLPQ	0.70	62.50	954.54
P146	VLTKEIYAK	0.08	55.56	1064.64
P147	DDIKKVV	1.68	42.86	816.48
P148	IPDGEKVDFD	0.42	40.00	1134.53
P149	DKEIEDLK	0.54	25.00	989.51
P150	DKIKDAKII	1.38	44.44	1043.65
P151	VTEAKLVEVK	0.61	50.00	1115.67
P152	APKIPDGEKV	1.80	50.00	1053.59
P153	IGSLDVEKVK	0.14	40.00	1087.64
P154	IAANWIQQEK	1.01	50.00	1200.64
P155	SVVDRPDIKK	0.46	40.00	1156.67
P156	VYPWTQRH	0.29	50.00	1086.55
P157	VPIEETVETK	0.50	40.00	1144.61
P158	YKATEPVI	0.66	62.50	920.51
P159	IPVISVPEK	1.67	66.67	981.60
P160	FPEYDGKDR	0.28	33.33	1126.52
P161	IIGEDPKYL	0.15	55.56	1047.57
P162	PAVADADKK	0.67	55.56	914.49
P163	VDFNVPMK	0.17	62.50	949.48
P164	IPTDITK	0.05	42.86	787.46
P165	IPIIGKPTPK	1.26	60.00	1063.69
P166	VVDADEMYLK	0.38	60.00	1182.57
P167	NPIALKDS	0.20	50.00	857.47
P168	GEPSITKVTR	0.62	30.00	1087.61
P169	VDRPDIKKAT	0.62	40.00	1142.65
P170	GIEIPTEK	0.14	37.50	886.49
P171	TPFGGFDK	0.51	37.50	868.42
P172	DADEMYVKGK	0.53	40.00	1155.54
P173	EFAVMVKG	0.18	62.50	880.46
P174	EPEGIKSPPR	0.61	40.00	1109.59
P175	IKEDAISVLK	0.59	50.00	1115.67
P176	WSLINKQA	0.22	50.00	959.53
P177	DAIPIVAAK	0.41	77.78	897.54
P178	DFTEPPKF	0.47	50.00	980.47
P179	AKVLTKEIY	0.08	55.56	1064.64

P180	IHVTMVETTK	0.51	40.00	1158.62
P181	ALPVKDPVVD	0.78	70.00	1052.60
P182	EIVRDIKEKL	0.96	40.00	1242.74
P183	IDPEGTFKY	0.43	44.44	1069.52
P184	YEITWSDDKK	0.88	30.00	1284.61
P185	VISVPEK	1.48	57.14	771.46
P186	VAYWRQAGLS	0.22	60.00	1150.60
P187	HEKYDKSLPV	0.47	40.00	1215.64
P188	DPEGTFKY	0.07	37.50	956.44
P189	TVFADDDDKK	0.73	30.00	1153.54
P190	KVDFDDIQKK	1.37	30.00	1235.66
P191	FVATKGGVQP	0.35	50.00	1003.56
P192	HDFTEPPK	0.56	37.50	970.46
P193	IALKDRIEK	0.98	44.44	1085.67
P194	SPMPVIAH	0.48	75.00	851.44
P195	DELDKKVRLS	0.16	30.00	1202.67
P196	DELDKKVRL	0.15	33.33	1115.64
P197	TGILKPGMV	0.01	55.56	915.53
P198	FTLPPHNSR	0.03	44.44	1068.56
P199	EIHVSIDK	0.01	37.50	940.51
P200	EHDFTEPPKF	0.24	40.00	1246.57
P201	GPVMPIKT	1.29	62.50	842.48
P202	SLQDDPLLVH	0.09	50.00	1136.59
P203	DKIKDAKIIF	1.46	50.00	1190.71
P204	NDDPKILH	0.34	37.50	951.49
P205	KPTPPTGNLQ	1.40	40.00	1052.57
P206	IAITEDKP	0.47	50.00	886.49
P207	AIDSMIPAQK	0.29	60.00	1073.57
P208	DFPEYDGKDR	0.12	30.00	1241.54
P209	IPVVDDKFT	0.67	55.56	1033.56
P210	EDTKEVTVKP	0.10	30.00	1145.60
P211	LPVEVAK	0.86	71.43	755.47
P212	SAFKVLDPEG	0.43	50.00	1062.55
P213	IKKGEEIRID	0.41	30.00	1200.69
P214	GILKPGMV	0.00	62.50	814.49
P215	ELMRPVSEL	0.41	55.56	1073.57
P216	DMEIKHFKPL	0.10	50.00	1257.67
P217	NPPKYDKIE	1.51	44.44	1103.57
P218	SMIPAQK	0.59	57.14	774.42
P219	VADADKK	0.80	42.86	746.40
P220	PTKVPKAE	0.03	50.00	869.51
P221	EDEPELR	0.95	28.57	887.41
P222	IPVVDDKFTL	0.10	60.00	1146.64
P223	LVFPSEIVGK	0.36	60.00	1088.64
P224	PIQIGEHLP	0.45	55.56	1003.56
P225	LPSLPKF	0.78	71.43	801.49
P226	TEKEQIVP	0.68	37.50	943.51

P227	SKKIGDIIYK	1.03	40.00	1164.70
P228	EIDPEGTFKY	0.17	40.00	1198.56
P229	PEGIKSPPR	0.31	44.44	980.55
P230	EAYKNISNLD	0.28	40.00	1166.57
P231	TGVKEVVEAA	0.22	50.00	1002.55
P232	GPVMPIKTVH	1.03	60.00	1078.61
P233	ILKDPDL	0.21	57.14	813.47
P234	IEAPPHIF	1.17	75.00	923.50
P235	DIAKGYDMKS	0.82	40.00	1127.54
P236	DIDIRKDLYA	0.66	50.00	1221.65
P237	DRVHIVIDK	0.22	44.44	1094.63
P238	DFDDIQKKR	0.22	22.22	1164.60
P239	TPTGWKFFGN	0.70	40.00	1154.56
P240	GDIIYKM	0.02	57.14	839.43
P241	IIAPPERKYS	0.81	60.00	1173.66
P242	LFDKPVS	0.60	57.14	805.45
P243	DTPEILRVK	0.22	44.44	1070.62
P244	KRHDDPVEYF	0.46	40.00	1305.62
P245	DWDINLK	0.28	42.86	903.46
P246	EEPFGGFK	0.31	37.50	910.43
P247	ATEVSKLT	0.33	37.50	848.47
P248	DAPEIVLAK	0.57	66.67	955.55
P249	GFGKFTL	0.07	42.86	769.42
P250	SFNPIALKD	0.17	55.56	1004.54
P251	PQTKTYFS	0.40	37.50	971.48
P252	QTIINSPNK	1.18	33.33	1014.56
P253	GHHVKEDAIP	1.15	40.00	1102.56
P254	GDFTPEIH	0.13	37.50	915.42
P255	DAVYKEDLK	0.73	44.44	1080.56
P256	IAKADVSKGF	0.34	50.00	1035.58
P257	LTVFPQTKTY	0.04	50.00	1197.65
P258	VIPVVDDKFT	0.33	60.00	1132.62
P259	PFGGFDK	0.18	42.86	767.37
P260	DIKGPDF	0.44	42.86	791.39
P261	QKIEEIFKKH	0.17	30.00	1299.74
P262	GEPSITKVT	1.00	33.33	931.51
P263	TVFPQTK	0.33	42.86	820.46
P264	EIEDLKIK	0.25	37.50	987.57
P265	KVLPGVDALS	0.23	60.00	998.59
P266	TPTPVRPV	0.73	62.50	866.51
P267	PVISVPEKK	2.08	55.56	996.61
P268	DFDDIQKK	0.73	25.00	1008.50
P269	TEVPEVPKVK	1.29	50.00	1125.65
P270	IKEGDIVKRT	0.32	30.00	1158.68
P271	PIKVPESDDL	1.36	50.00	1112.58
P272	PQYSDLEK	0.81	37.50	979.47

P273	KIKIIAPPER	1.52	60.00	1164.75
P274	IDPEGTFKYI	0.30	50.00	1182.60
P275	YETPTGWKF	0.35	44.44	1128.54
P276	TVGYSTKL	0.04	37.50	868.48
P277	HDFTEPPKF	0.48	44.44	1117.53
P278	DTPDIRQVK	0.19	33.33	1071.58
P279	HIELPFKGKP	0.55	50.00	1165.67
P280	AKYDDIKKV	2.57	44.44	1079.61
P281	IAMYEHKIF	0.94	66.67	1151.59
P282	LFDKPVSPLL	0.96	70.00	1128.67
P283	VVVSALGKQY	0.96	60.00	1063.61
P284	KRIEAPPHIF	1.79	60.00	1207.69
P285	PSIVHRK	0.32	42.86	836.51
P286	EIEDLKIKV	0.65	44.44	1086.64
P287	RHDDPVEYF	0.53	44.44	1177.53
P288	EPTVIDEVR	0.34	44.44	1057.55
P289	LPEGFESSHH	0.21	30.00	1139.51
P290	TDTGKTVTVK	0.20	20.00	1049.58
P291	EIPEVPEVYR	1.58	60.00	1230.64
P292	VFKDLLDPI	0.29	66.67	1059.61
P293	IVYPWTQRHF	0.74	60.00	1346.70
P294	KEIEDLKIK	0.47	33.33	1115.67
P295	IGPKYNELR	0.91	44.44	1089.61
P296	AAIYLRKP	0.61	75.00	931.57
P297	WDDMEKIWHH	0.46	40.00	1396.61
P298	KEDAISVLK	0.81	44.44	1002.58
P299	LTRELGLTEE	0.08	30.00	1160.62
P300	SIKGFTLPPH	0.66	50.00	1096.61
P301	APLAKVIHDN	0.07	60.00	1077.61
P302	EDQELLRPH	0.83	33.33	1136.57
P303	VPPFTFHVK	1.54	66.67	1071.60
P304	IPEVPEVYR	1.71	66.67	1101.59
P305	APIRIPVGP	1.10	77.78	919.57
P306	RVDPANFKIL	0.37	60.00	1172.68
P307	DTDFEHRLP	0.23	33.33	1129.53
P308	ADKIKDAKII	1.48	50.00	1114.68
P309	DILDKKIEK	0.94	33.33	1101.65
P310	VIEHIQVNK	1.09	44.44	1079.62
P311	IIGDDPKF	0.27	50.00	904.48
P312	LEDIKL	0.11	50.00	730.43
P313	GPSIVHRK	0.20	37.50	893.53
P314	KDELDKKVRL	0.12	30.00	1243.74
P315	DKIKDAKI	1.54	37.50	930.56
P316	TPEVHVSIDK	0.25	40.00	1124.59
P317	DSGALPVKDP	0.54	50.00	998.52
P318	IGEKLDERE	0.04	22.22	1088.56
P319	DAPRAVFPSI	0.13	70.00	1072.58

P320	FLFDKPVSPL	1.06	70.00	1162.65
P321	EIDEEPVSKA	0.66	40.00	1116.54
P322	LDHDVQEDKI	0.31	30.00	1211.59
P323	IKIIAPPERK	1.52	60.00	1164.75
P324	KLIGHVM	0.27	57.14	797.47
P325	EELFNLR	0.58	42.86	920.48
P326	THKPIEVKGP	1.70	40.00	1105.64
P327	WRKNIEDK	0.64	25.00	1088.58
P328	EDLKVDKVIK	1.58	40.00	1186.70
P329	IGSMDVEKAK	0.27	40.00	1077.56
P330	ADILKDLK	0.13	50.00	915.55
P331	ADKEIEDLKI	0.81	40.00	1173.64
P332	DLKVDKVIK	1.71	44.44	1057.66
P333	VPEPEKPAP	0.80	66.67	963.51
P334	DPEGTFKYI	0.43	44.44	1069.52
P335	EIPSVAVTK	0.57	55.56	943.55
P336	VDADEMYLK	0.19	55.56	1083.50
P337	ESVDVIKVK	0.87	44.44	1016.60
P338	KADKEIEDLK	0.56	30.00	1188.65
P339	KIIAPPERKY	1.81	60.00	1214.73
P340	LDHDVQEDK	0.13	22.22	1098.51
P341	YEAFVKHIM	0.52	66.67	1137.58
P342	YPTEDVPRKL	0.78	50.00	1217.65
P343	DLTVRLE	0.25	42.86	845.47
P344	VKHIMSV	0.04	57.14	813.47
P345	IDKVDEERYD	0.60	30.00	1281.60
P346	EIASDVKYK	1.63	44.44	1052.56
P347	EAMDVKVVK	1.30	55.56	1018.56
P348	LEVTVKDL	0.22	50.00	916.53
P349	PGQQKTDDK	0.20	11.11	1016.50
P350	DDIKKVVK	1.83	37.50	944.58
P351	IPTDITKN	0.05	37.50	901.50
P352	KIKDAKII	1.20	50.00	928.62
P353	LTETHEVVTR	1.08	30.00	1184.63
P354	SLPVEVAK	1.58	62.50	842.50
P355	LRDWHHDR	0.60	25.00	1134.56
P356	DSVDHVKVKK	0.82	30.00	1154.65
