# Supplementary material for: Formation and inhibition mechanism of novel angiotensin I converting enzyme inhibitory peptides from Chouguiyu
Source: Front Nutr. 2022 Jul 22;9:920945. doi: 10.3389/fnut.2022.920945 (PMC9355153; doi:10.3389/fnut.2022.920945)
Supplement: Supplementary file 5 [file Data_Sheet_5.PDF]

Table S2 Cleavage locations in precursor proteins of ACE inhibitory peptides from *Chouguiyu*

Peptide number	Hit name	Protein length	Start position	End position	Precursor protein	Description of precursor protein
P1	XP_010746091.1	247	240	247	Pro37	Triosephosphate isomerase
P2	XP_028274893.1	109	103	109	Pro38	Parvalbumin
P3	XP_028274893.1	171	153	161	Pro3	Troponin I
P4	BBA68528.1	143	135	143	Pro12	14 kDa phosphohistidine phosphatase-like
P5	XP_026208698.1	84	5	13	Pro19	Myosin heavy chain, fast skeletal muscle
P6	XP_028274893.1	143	35	43	Pro25	Hemoglobin subunit alpha-B-like
P7	XP_026181823.1	212	105	111	Pro8	Troponin T, fast skeletal muscle isoforms-like isoform X2
P8	BBA68528.1	491	424	430	Pro4	Myosin-binding protein H-like
P9	KAF3694312.1	633	364	371	Pro26	Nebulin isoform X8
P10	TSK31301.1	411	60	69	Pro1	Muscle-type creatine kinase
P11	XP_033469419.1	212	24	33	Pro27	Troponin T, fast skeletal muscle isoforms-like isoform X7
P12	ACM07327.1	212	26	33	Pro27	Troponin T, fast skeletal muscle isoforms-like isoform X7
P13	XP_022620269.1	80	50	57	Pro10	Actin, cytoplasmic 1
P14	TSK31301.1	566	211	218	Pro9	Nebulin
P15	KAF1382423.1	124	1	6	Pro52	Synuclein-like isoform X1
P16	KAA8591518.1	171	152	161	Pro3	Troponin I
P17	XP_010728431.1	212	102	111	Pro8	Troponin T, fast skeletal muscle isoforms-like isoform X2
P18	ABO31103.1	212	26	35	Pro27	Troponin T, fast skeletal muscle isoforms-like isoform X7
P19	XP_028274893.1	385	109	118	Pro6	Hypothetical protein OJAV_G00075610
P20	XP_023149595.1	2517	1648	1654	Pro2	Nebulin
P21	TKS68253.1	343	268	277	Pro7	Glyceraldehyde-3-phosphate dehydrogenase
P22	XP_008295970.1	343	266	275	Pro7	Glyceraldehyde-3-phosphate dehydrogenase
P23	XP_030284270.1	385	110	118	Pro6	Hypothetical protein OJAV_G00075610
P24	XP_022620269.1	343	267	275	Pro7	Glyceraldehyde-3-phosphate dehydrogenase
P25	XP_008295970.1	1547	1321	1330	Pro15	Titin-like
P26	AKA66317.1	491	379	385	Pro4	Myosin-binding protein H-like
P27	XP_028274893.1	193	154	163	Pro11	Adenylate kinase isoenzyme 1
P28	ACM07327.1	212	92	99	Pro8	Troponin T, fast skeletal muscle isoforms-like isoform X2
P29	TKS65323.1	7374	7038	7046	Pro20	Titin-like

P30	XP_030284270.1	411	61	69	Pro1	Muscle-type creatine kinase
P31	KAF1379223.1	411	194	201	Pro1	Muscle-type creatine kinase
P32	XP_030281795.1	343	268	275	Pro7	Glyceraldehyde-3-phosphate dehydrogenase
P33	XP_028274893.1	491	377	385	Pro4	Myosin-binding protein H-like
P34	XP_026208698.1	212	104	111	Pro8	Troponin T, fast skeletal muscle isoforms-like isoform X2
P35	TSK31301.1	212	25	33	Pro27	Troponin T, fast skeletal muscle isoforms-like isoform X7
P36	XP_018522973.1	1107	1048	1054	Pro22	Myosin-binding protein C, fast-type-like isoform X7
P37	XP_028274893.1	566	456	462	Pro9	Nebulin
P38	ACM07327.1	112	53	61	Pro39	Skeletal alpha-actin
P39	KAA8591518.1	147	34	42	Pro21	Hemoglobin beta-A chain
P40	XP_018533020.1	1547	1282	1291	Pro15	Titin-like
P41	XP_029371681.1	212	103	111	Pro8	Troponin T, fast skeletal muscle isoforms-like isoform X2
P42	XP_022595156.1	1107	1048	1055	Pro22	Myosin-binding protein C, fast-type-like isoform X7
P43	XP_028278608.1	633	364	372	Pro26	Nebulin isoform X8
P44	XP_022595843.1	80	37	46	Pro10	Actin, cytoplasmic 1
P45	XP_028437696.1	143	35	42	Pro25	Hemoglobin subunit alpha-B-like
P46	XP_022595843.1	270	36	43	Pro53	Collagen alpha-1(I) chain-like isoform X1
P47	XP_020511666.1	1275	16	21	Pro5	Fast skeletal muscle myosin heavy chain isoform 3
P48	TSK31301.1	212	92	100	Pro8	Troponin T, fast skeletal muscle isoforms-like isoform X2
P49	KAF1382423.1	290	185	193	Pro16	LIM domain-binding protein 3-like isoform X2
P50	XP_023124481.1	188	41	50	Pro54	Inosine triphosphate pyrophosphatase
P51	XP_028278608.1	2517	812	821	Pro2	Nebulin
P52	XP_028278608.1	633	365	371	Pro26	Nebulin isoform X8
P53	XP_028274893.1	385	317	323	Pro6	Hypothetical protein OJAV_G00075610
P54	TKS65323.1	1275	14	21	Pro5	Fast skeletal muscle myosin heavy chain isoform 3
P55	XP_030281795.1	80	48	57	Pro10	Actin, cytoplasmic 1
P56	ACM07327.1	411	32	40	Pro1	Muscle-type creatine kinase
P57	XP_023125996.1	344	225	234	Pro17	Glyceraldehyde 3-phosphate dehydrogenase isoform 2
P58	XP_029371681.1	343	267	276	Pro7	Glyceraldehyde-3-phosphate dehydrogenase
P59	XP_028437696.1	171	86	95	Pro3	Troponin I
P60	XP_030281795.1	411	374	381	Pro1	Muscle-type creatine kinase
P61	XP_030284270.1	411	15	23	Pro1	Muscle-type creatine kinase
P62	ACM07327.1	343	268	276	Pro7	Glyceraldehyde-3-phosphate dehydrogenase

P63	XP_030284270.1	411	32	39	Pro1	Muscle-type creatine kinase
P64	AKA66317.1	1275	151	157	Pro5	Fast skeletal muscle myosin heavy chain isoform 3
P65	XP_031162439.1	1107	997	1005	Pro22	Myosin-binding protein C, fast-type-like isoform X7
P66	XP_029371681.1	566	698	706	Pro9	Nebulin
P67	XP_031162439.1	411	61	70	Pro1	Muscle-type creatine kinase
P68	ACM07327.1	143	35	42	Pro13	Hemoglobin subunit alpha-A
P69	XP_018533020.1	344	226	232	Pro17	Glyceraldehyde 3-phosphate dehydrogenase isoform 2
P70	XP_023149595.1	222	187	196	Pro23	Myosin light chain 1
P71	XP_010728431.1	344	226	234	Pro17	Glyceraldehyde 3-phosphate dehydrogenase isoform 2
P72	XP_023117637.1	2517	2275	2281	Pro2	Nebulin
P73	XP_022612894.1	255	222	231	Pro55	Phosphoglycerate mutase 2
P74	XP_022612894.1	190	168	176	Pro28	Myosin regulatory light chain 2, skeletal muscle isoform type 2-like
P75	ABO31103.1	147	33	42	Pro21	Hemoglobin beta-A chain
P76	XP_018531065.1	143	35	43	Pro13	Hemoglobin subunit alpha-A
P77	XP_018520797.1	1275	15	21	Pro5	Fast skeletal muscle myosin heavy chain isoform 3
P78	XP_030284270.1	224	102	111	Pro30	Actin, aortic smooth muscle
P79	XP_022620269.1	411	259	267	Pro1	Muscle-type creatine kinase
P80	KAF1386865.1	1275	74	83	Pro5	Fast skeletal muscle myosin heavy chain isoform 3
P81	TKS68253.1	411	193	201	Pro1	Muscle-type creatine kinase
P82	XP_026208698.1	2517	810	819	Pro2	Nebulin
P83	ACM07328.1	385	120	127	Pro6	Hypothetical protein OJAV_G00075610
P84	XP_018518739.1	190	131	140	Pro28	Myosin regulatory light chain 2, skeletal muscle isoform type 2-like
P85	XP_030284270.1	2517	1647	1654	Pro2	Nebulin
P86	TMS01143.1	313	251	260	Pro31	Hypothetical protein EPR50_G00074040
P87	XP_022595843.1	171	85	93	Pro3	Troponin I
P88	XP_010728431.1	1912	1092	1101	Pro14	Titin
P89	XP_028274893.1	221	196	202	Pro56	Heat shock protein beta-1
P90	XP_022620269.1	313	95	102	Pro29	Nascent polypeptide-associated complex subunit alpha
P91	XP_018538749.1	8008	291	298	Pro18	Titin-like
P92	XP_018531065.1	344	227	234	Pro17	Glyceraldehyde 3-phosphate dehydrogenase isoform 2
P93	KAF1379223.1	80	38	47	Pro10	Actin, cytoplasmic 1
P94	XP_018522973.1	80	49	57	Pro10	Actin, cytoplasmic 1

P95	XP_018522973.1	491	376	384	Pro4	Myosin-binding protein H-like
P96	XP_031162439.1	84	6	13	Pro19	Myosin heavy chain, fast skeletal muscle
P97	XP_022058184.1	491	472	481	Pro4	Myosin-binding protein H-like
P98	ADH43626.1	1275	17	26	Pro5	Fast skeletal muscle myosin heavy chain isoform 3
P99	XP_018522973.1	2517	70	79	Pro32	Nebulin
P100	XP_020481559.1	411	193	200	Pro1	Muscle-type creatine kinase
P101	KAF1379223.1	224	104	111	Pro30	Actin, aortic smooth muscle
P102	XP_022620269.1	84	7	13	Pro19	Myosin heavy chain, fast skeletal muscle
P103	XP_018550785.1	411	139	148	Pro1	Muscle-type creatine kinase
P104	XP_010728431.1	190	93	102	Pro28	Myosin regulatory light chain 2, skeletal muscle isoform type 2-like
P105	XP_022620269.1	224	98	107	Pro30	Actin, aortic smooth muscle
P106	XP_018531065.1	262	101	110	Pro57	Titin-like
P107	XP_008295970.1	80	50	56	Pro10	Actin, cytoplasmic 1
P108	XP_008295970.1	253	245	251	Pro58	Glutamine amidotransferase-like class 1 domain-containing protein 3A, mitochondrial
P109	AMN92722.1	171	153	162	Pro3	Troponin I
P110	XP_028274893.1	80	64	72	Pro10	Actin, cytoplasmic 1
P111	XP_022595156.1	222	187	195	Pro23	Myosin light chain 1
P112	ACM07328.1	344	226	233	Pro17	Glyceraldehyde 3-phosphate dehydrogenase isoform 2
P113	XP_030284270.1	491	378	387	Pro4	Myosin-binding protein H-like
P114	XP_010728431.1	1275	406	415	Pro5	Fast skeletal muscle myosin heavy chain isoform 3
P115	ATE88086.1	411	83	91	Pro1	Muscle-type creatine kinase
P116	XP_022595843.1	1107	997	1004	Pro22	Myosin-binding protein C, fast-type-like isoform X7
P117	XP_029371681.1	132	136	144	Pro24	Myozenin-2 calsarcin-1
P118	XP_028446506.1	1912	822	829	Pro14	Titin
P119	XP_018558729.1	2517	1437	1445	Pro2	Nebulin
P120	TWW63482.1	1912	1121	1129	Pro14	Titin
P121	XP_028274893.1	147	138	147	Pro21	Hemoglobin beta-A chain
P122	XP_018533020.1	411	32	41	Pro1	Muscle-type creatine kinase
P123	XP_030284270.1	171	146	155	Pro3	Troponin I
P124	TKS65323.1	343	269	278	Pro7	Glyceraldehyde-3-phosphate dehydrogenase
P125	XP_028462230.1	2517	1628	1637	Pro2	Nebulin
P126	XP_028437696.1	385	109	117	Pro6	Hypothetical protein OJAV_G00075610

P127	XP_026181823.1	87	17	24	Pro33	Troponin T, cardiac muscle isoforms-like
P128	XP_023124481.1	411	62	69	Pro1	Muscle-type creatine kinase
P129	AMN92722.1	84	66	75	Pro19	Myosin heavy chain, fast skeletal muscle
P130	XP_018518739.1	143	137	143	Pro12	14 kDa phosphohistidine phosphatase-like
P131	XP_008295970.1	411	36	42	Pro1	Muscle-type creatine kinase
P132	ABO31103.1	171	86	94	Pro3	Troponin I
P133	XP_028274893.1	344	225	233	Pro17	Glyceraldehyde 3-phosphate dehydrogenase isoform 2
P134	XP_010728431.1	377	26	32	Pro34	Calsequestrin-1-like
P135	TKS65323.1	193	184	192	Pro11	Adenylate kinase isoenzyme 1
P136	XP_031162439.1	143	38	46	Pro13	Hemoglobin subunit alpha-A
P137	KAF1383754.1	452	79	87	Pro35	LIM domain-binding protein 3 isoform X5
P138	XP_030284270.1	566	651	660	Pro9	Nebulin
P139	XP_006626442.1	313	95	103	Pro29	Nascent polypeptide-associated complex subunit alpha
P140	XP_018547633.1	149	109	118	Pro59	Dnaj homolog subfamily B member 1-like
P141	XP_018550785.1	80	39	47	Pro10	Actin, cytoplasmic 1
P142	KAF1378016.1	222	188	196	Pro23	Myosin light chain 1
P143	XP_030284270.1	8008	3472	3478	Pro18	Titin-like
P144	XP_018522973.1	452	275	283	Pro35	LIM domain-binding protein 3 isoform X5
P145	XP_018518739.1	452	276	283	Pro35	LIM domain-binding protein 3 isoform X5
P146	KAF1379223.1	411	33	41	Pro1	Muscle-type creatine kinase
P147	XP_030284270.1	343	269	275	Pro7	Glyceraldehyde-3-phosphate dehydrogenase
P148	XP_029282430.1	87	20	29	Pro33	Troponin T, cardiac muscle isoforms-like
P149	ATE88086.1	171	86	93	Pro3	Troponin I
P150	TKS68253.1	193	3	11	Pro11	Adenylate kinase isoenzyme 1
P151	ABO31103.1	313	119	128	Pro29	Nascent polypeptide-associated complex subunit alpha
P152	XP_022595843.1	87	17	26	Pro33	Troponin T, cardiac muscle isoforms-like
P153	XP_019751440.1	2517	315	324	Pro32	Nebulin
P154	XP_023124481.1	171	21	30	Pro3	Troponin I
P155	XP_022620269.1	2517	2419	2428	Pro2	Nebulin
P156	XP_030289355.1	147	35	42	Pro21	Hemoglobin beta-A chain
P157	TKS65323.1	1547	1266	1275	Pro15	Titin-like
P158	XP_030284270.1	193	154	161	Pro11	Adenylate kinase isoenzyme 1
P159	KAF1379223.1	1547	1321	1329	Pro15	Titin-like
P160	XP_028274893.1	377	26	34	Pro34	Calsequestrin-1-like

P161	XP_030284270.1	1107	1047	1055	Pro22	Myosin-binding protein C, fast-type-like isoform X7
P162	XP_031137990.1	491	476	484	Pro4	Myosin-binding protein H-like
P163	XP_023272534.1	416	23	30	Pro40	Phosphoglycerate kinase 1
P164	XP_023124481.1	5469	1020	1026	Pro36	Titin
P165	XP_030284270.1	5469	51	60	Pro36	Titin
P166	XP_022595843.1	1275	40	49	Pro5	Fast skeletal muscle myosin heavy chain isoform 3
P167	XP_030284270.1	290	124	131	Pro16	LIM domain-binding protein 3-like isoform X2
P168	XP_031162439.1	793	692	701	Pro41	Periostin-like isoform X1
P169	XP_018531065.1	2517	2421	2430	Pro2	Nebulin
P170	XP_022595156.1	546	310	317	Pro60	Pyruvate kinase PKM-like
P171	KAF1382423.1	132	73	80	Pro24	Myozenin-2 calsarcin-1
P172	XP_022595843.1	84	34	43	Pro19	Myosin heavy chain, fast skeletal muscle
P173	XP_022595156.1	109	102	109	Pro38	Parvalbumin
P174	XP_030284270.1	7374	7038	7047	Pro20	Titin-like
P175	XP_029371681.1	2517	671	680	Pro2	Nebulin
P176	XP_022595156.1	103	76	83	Pro61	Hypothetical protein E3U43_021098
P177	XP_030284270.1	566	418	426	Pro9	Nebulin
P178	TKS68253.1	491	379	386	Pro4	Myosin-binding protein H-like
P179	XP_010728431.1	411	31	39	Pro1	Muscle-type creatine kinase
P180	XP_023272534.1	308	214	223	Pro62	Plectin-like isoform X3
P181	XP_028274893.1	290	184	193	Pro16	LIM domain-binding protein 3-like isoform X2
P182	XP_018520797.1	385	236	245	Pro6	Hypothetical protein OJAV_G00075610
P183	XP_022595156.1	143	37	45	Pro12	14 kDa phosphohistidine phosphatase-like
P184	ACM07327.1	143	134	143	Pro12	14 kDa phosphohistidine phosphatase-like
P185	XP_023117637.1	1547	1323	1329	Pro15	Titin-like
P186	XP_029282430.1	50	2	11	Pro63	ATP synthase subunit epsilon, mitochondrial
P187	XP_031162439.1	333	135	144	Pro64	Glyceraldehyde 3-phosphate dehydrogenase isoform 1
P188	XP_022620269.1	143	38	45	Pro12	14 kDa phosphohistidine phosphatase-like
P189	XP_031703795.1	94	13	22	Pro65	Endoplasmic reticulum chaperone bip
P190	XP_029371681.1	212	103	112	Pro8	Troponin T, fast skeletal muscle isoforms-like isoform X2
P191	XP_029016693.1	229	212	221	Pro42	Synaptopodin 2-like protein
P192	TKS68253.1	491	378	385	Pro4	Myosin-binding protein H-like
P193	XP_018550968.1	212	140	148	Pro8	Troponin T, fast skeletal muscle isoforms-like isoform X2

P194	KAF1379223.1	290	99	106	Pro16	LIM domain-binding protein 3-like isoform X2
P195	XP_018522973.1	1912	734	743	Pro14	Titin
P196	XP_030289355.1	1912	734	742	Pro14	Titin
P197	XP_010728431.1	461	269	277	Pro43	Elongation factor 1-alpha
P198	XP_010728431.1	411	140	148	Pro1	Muscle-type creatine kinase
P199	XP_030284270.1	143	122	129	Pro13	Hemoglobin subunit alpha-A
P200	XP_018518739.1	491	377	386	Pro4	Myosin-binding protein H-like
P201	XP_022620269.1	211	152	159	Pro44	Ribosomal protein L13
P202	TKS65323.1	566	209	218	Pro9	Nebulin
P203	XP_020501769.2	193	3	12	Pro11	Adenylate kinase isoenzyme 1
P204	XP_028274893.1	633	365	372	Pro26	Nebulin isoform X8
P205	AKA66317.1	315	231	240	Pro66	PDZ and LIM domain protein 3
P206	ABO31103.1	48	14	21	Pro67	Protein NDRG2
P207	KAF1379223.1	411	372	381	Pro1	Muscle-type creatine kinase
P208	ACM07327.1	377	25	34	Pro34	Calsequestrin-1-like
P209	XP_031137990.1	2517	2275	2283	Pro2	Nebulin
P210	XP_028289547.1	93	22	31	Pro68	Butyrophilin subfamily 2 member A1-like
P211	XP_031174735.1	313	293	299	Pro31	Hypothetical protein EPR50_G00074040
P212	XP_018518739.1	190	124	133	Pro28	Myosin regulatory light chain 2, skeletal muscle isoform type 2-like
P213	ABO31103.1	8008	6666	6675	Pro18	Titin-like
P214	XP_023124481.1	461	270	277	Pro43	Elongation factor 1-alpha
P215	XP_028274893.1	7374	6387	6395	Pro20	Titin-like
P216	AKA66317.1	1912	821	830	Pro14	Titin
P217	ACM07327.1	1275	81	89	Pro5	Fast skeletal muscle myosin heavy chain isoform 3
P218	TDH12775.1	411	375	381	Pro1	Muscle-type creatine kinase
P219	XP_026208698.1	491	478	484	Pro4	Myosin-binding protein H-like
P220	KAF1379223.1	1547	1284	1291	Pro15	Titin-like
P221	XP_030284270.1	185	149	155	Pro69	Putative obscurin-like
P222	AGA12056.1	2517	2275	2284	Pro2	Nebulin
P223	ACM07327.1	167	133	142	Pro70	40S ribosomal protein S7
P224	XP_028274893.1	190	30	38	Pro71	Peroxiredoxin-5, mitochondrial
P225	XP_028274893.1	229	147	153	Pro42	Synaptopodin 2-like protein
P226	KAA8591518.1	191	158	165	Pro45	Hypothetical protein EPR50_G00173570

P227	XP_008295970.1	2517	2149	2158	Pro2	Nebulin
P228	XP_018558111.1	143	36	45	Pro12	14 kDa phosphohistidine phosphatase-like
P229	XP_023124481.1	7374	7039	7047	Pro20	Titin-like
P230	XP_030266024.1	2517	2218	2227	Pro2	Nebulin
P231	XP_026179890.1	921	355	364	Pro46	Titin-like
P232	XP_027134606.1	211	152	161	Pro44	Ribosomal protein L13
P233	XP_029371681.1	132	39	45	Pro24	Myozenin-2 calsarcin-1
P234	XP_024115186.1	1275	149	156	Pro5	Fast skeletal muscle myosin heavy chain isoform 3
P235	XP_028274893.1	566	652	661	Pro9	Nebulin
P236	KAF1378016.1	112	53	62	Pro39	Skeletal alpha-actin
P237	XP_010728431.1	8008	1801	1809	Pro18	Titin-like
P238	CBN81678.1	212	105	113	Pro8	Troponin T, fast skeletal muscle isoforms-like isoform X2
P239	KAF1379223.1	581	380	389	Pro47	Phosphoglucomutase-1
P240	XP_008295970.1	2517	2153	2159	Pro2	Nebulin
P241	XP_029371681.1	385	358	367	Pro6	Hypothetical protein OJAV_G00075610
P242	XP_026179890.1	411	193	199	Pro1	Muscle-type creatine kinase
P243	XP_018531065.1	2517	1544	1552	Pro2	Nebulin
P244	XP_010746091.1	100	91	100	Pro48	Cystatin-B-like
P245	TSK31301.1	248	52	58	Pro49	Neuroblast differentiation-associated protein AHNAK
P246	TKS65323.1	8008	3694	3701	Pro18	Titin-like
P247	XP_028274893.1	2517	2464	2471	Pro2	Nebulin
P248	XP_028437696.1	566	138	146	Pro9	Nebulin
P249	XP_011475514.1	2517	1081	1087	Pro2	Nebulin
P250	KAF1379223.1	290	122	130	Pro16	LIM domain-binding protein 3-like isoform X2
P251	TDH09329.1	143	38	45	Pro13	Hemoglobin subunit alpha-A
P252	KAA8587284.1	222	145	153	Pro23	Myosin light chain 1
P253	XP_018531065.1	566	412	421	Pro9	Nebulin
P254	ACM07327.1	143	117	124	Pro13	Hemoglobin subunit alpha-A
P255	XP_030279017.1	2517	297	305	Pro32	Nebulin
P256	XP_022595843.1	193	81	90	Pro11	Adenylate kinase isoenzyme 1
P257	XP_010728431.1	143	34	43	Pro13	Hemoglobin subunit alpha-A
P258	XP_010740496.2	2517	2274	2283	Pro2	Nebulin
P259	ACM07327.1	132	74	80	Pro24	Myozenin-2 calsarcin-1

P260	XP_030279017.1	248	137	143	Pro49	Neuroblast differentiation-associated protein AHNAK
P261	XP_018550118.1	411	258	267	Pro1	Muscle-type creatine kinase
P262	XP_018522973.1	793	692	700	Pro41	Periostin-like isoform X1
P263	TKS79294.1	143	35	41	Pro13	Hemoglobin subunit alpha-A
P264	ABO31103.1	171	88	95	Pro3	Troponin I
P265	XP_030284270.1	416	406	415	Pro40	Phosphoglycerate kinase 1
P266	ACM07327.1	921	292	299	Pro46	Titin-like
P267	KAF1379223.1	1547	1322	1330	Pro15	Titin-like
P268	XP_022620269.1	212	105	112	Pro8	Troponin T, fast skeletal muscle isoforms-like isoform X2
P269	XP_011475514.1	599	124	133	Pro72	Trichohyalin-like
P270	XP_008281116.1	551	124	133	Pro73	ATP synthase subunit alpha, mitochondrial-like
P271	TDH13026.1	132	135	144	Pro24	Myozenin-2 calsarcin-1
P272	TKS65323.1	103	2	9	Pro74	Protein S100-A14-like
P273	KAF1382423.1	385	355	364	Pro6	Hypothetical protein OJAV_G00075610
P274	ACM07327.1	143	37	46	Pro12	14 kDa phosphohistidine phosphatase-like
P275	XP_030284270.1	581	378	386	Pro47	Phosphoglucomutase-1
P276	XP_031149844.1	491	396	403	Pro4	Myosin-binding protein H-like
P277	ABO31103.1	491	378	386	Pro4	Myosin-binding protein H-like
P278	XP_008289652.1	2517	1722	1730	Pro2	Nebulin
P279	XP_022620269.1	7374	1783	1792	Pro20	Titin-like
P280	TKS65323.1	343	266	274	Pro7	Glyceraldehyde-3-phosphate dehydrogenase
P281	XP_022612894.1	566	504	512	Pro75	Glucose-6-phosphate isomerase
P282	XP_029371681.1	411	193	202	Pro1	Muscle-type creatine kinase
P283	XP_018518739.1	147	137	146	Pro21	Hemoglobin beta-A chain
P284	XP_028437696.1	1275	147	156	Pro5	Fast skeletal muscle myosin heavy chain isoform 3
P285	TDH07799.1	385	396	402	Pro6	Hypothetical protein OJAV_G00075610
P286	XP_030284270.1	171	88	96	Pro3	Troponin I
P287	XP_023149595.1	100	92	100	Pro48	Cystatin-B-like
P288	XP_022595843.1	449	85	93	Pro76	Tubulin alpha chain-like isoform X2
P289	XP_019751440.1	102	93	102	Pro77	Cytochrome c oxidase subunit 6A, mitochondrial
P290	AKA66317.1	1275	64	73	Pro5	Fast skeletal muscle myosin heavy chain isoform 3
P291	XP_018522973.1	8008	3469	3478	Pro18	Titin-like
P292	XP_031162439.1	411	84	92	Pro1	Muscle-type creatine kinase
P293	XP_022620269.1	147	34	43	Pro21	Hemoglobin beta-A chain

P294	XP_023149595.1	171	87	95	Pro3	Troponin I
P295	XP_028446506.1	215	193	201	Pro78	Hypothetical protein EPR50_G00051190
P296	TSK31301.1	84	5	12	Pro19	Myosin heavy chain, fast skeletal muscle
P297	XP_026179890.1	385	108	117	Pro6	Hypothetical protein OJAV_G00075610
P298	XP_018521732.1	2517	672	680	Pro2	Nebulin
P299	ABO31103.1	95	85	94	Pro79	D-dopachrome tautomerase
P300	XP_022620269.1	411	136	145	Pro1	Muscle-type creatine kinase
P301	XP_018531065.1	344	167	176	Pro17	Glyceraldehyde 3-phosphate dehydrogenase isoform 2
P302	XP_026208698.1	1912	1034	1042	Pro14	Titin
P303	XP_031167710.1	265	231	239	Pro80	Polymerase I and transcript release factor-like
P304	XP_030284270.1	8008	3470	3478	Pro18	Titin-like
P305	XP_028446506.1	517	119	127	Pro81	ATP synthase subunit beta, mitochondrial
P306	KAF1379223.1	143	94	103	Pro25	Hemoglobin subunit alpha-B-like
P307	ABO31103.1	812	762	770	Pro82	ATP-dependent 6-phosphofructokinase, muscle type-like isoform X1
P308	TSK31301.1	193	2	11	Pro11	Adenylate kinase isoenzyme 1
P309	XP_028274893.1	287	135	143	Pro83	Heat shock protein HSP 90-alpha 1
P310	KAA8591518.1	191	120	128	Pro45	Hypothetical protein EPR50_G00173570
P311	TKS65323.1	491	423	430	Pro4	Myosin-binding protein H-like
P312	ACM07327.1	175	119	124	Pro84	Ankyrin-3-like isoform X20
P313	XP_026179890.1	385	395	402	Pro6	Hypothetical protein OJAV_G00075610
P314	XP_028274893.1	1912	733	742	Pro14	Titin
P315	XP_028274893.1	193	3	10	Pro11	Adenylate kinase isoenzyme 1
P316	TKS65323.1	143	120	129	Pro25	Hemoglobin subunit alpha-B-like
P317	XP_028274893.1	290	181	190	Pro16	LIM domain-binding protein 3-like isoform X2
P318	ABO31103.1	247	126	134	Pro37	Triosephosphate isomerase
P319	XP_023149595.1	80	44	53	Pro10	Actin, cytoplasmic 1
P320	XP_022595843.1	411	192	201	Pro1	Muscle-type creatine kinase
P321	XP_028274893.1	313	250	259	Pro29	Nascent polypeptide-associated complex subunit alpha
P322	XP_022595156.1	84	29	38	Pro50	Uridine-cytidine kinase-like 1 isoform X2
P323	KAA8591518.1	385	356	365	Pro6	Hypothetical protein OJAV_G00075610
P324	XP_022620269.1	335	9	15	Pro85	Hypothetical protein EPR50_G00085720
P325	KAF1382423.1	152	130	136	Pro86	NADH dehydrogenase

P326	KAA8591518.1	290	135	144	Pro16	LIM domain-binding protein 3-like isoform X2
P327	XP_030284270.1	171	154	161	Pro3	Troponin I
P328	TKS84309.1	266	8	17	Pro51	Syntenin-1-like
P329	XP_026181823.1	566	557	566	Pro9	Nebulin
P330	AKA66317.1	170	115	122	Pro87	Leiomodin-2
P331	XP_022595156.1	171	85	94	Pro3	Troponin I
P332	ABO31103.1	266	9	17	Pro51	Syntenin-1-like
P333	BBA68528.1	221	137	145	Pro88	Reticulon-4-like isoform X1
P334	XP_028274893.1	143	38	46	Pro12	14 kDa phosphohistidine phosphatase-like
P335	XP_010728431.1	200	4	12	Pro89	Caveolae-associated protein 4a-like
P336	ABO31103.1	1275	41	49	Pro5	Fast skeletal muscle myosin heavy chain isoform 3
P337	XP_028274893.1	2517	813	821	Pro2	Nebulin
P338	XP_030284270.1	171	84	93	Pro3	Troponin I
P339	KAF1379223.1	385	357	366	Pro6	Hypothetical protein OJAV_G00075610
P340	XP_026181823.1	84	29	37	Pro50	Uridine-cytidine kinase-like 1 isoform X2
P341	XP_030284270.1	41	31	39	Pro90	Myosin light chain 3-like
P342	XP_028437696.1	259	87	96	Pro91	60S ribosomal protein L6
P343	ACM07327.1	343	257	263	Pro7	Glyceraldehyde-3-phosphate dehydrogenase
P344	XP_008295970.1	222	215	221	Pro23	Myosin light chain 1
P345	ATE45999.1	171	68	77	Pro3	Troponin I
P346	XP_028446506.1	2517	1173	1181	Pro2	Nebulin
P347	XP_028437696.1	1487	139	147	Pro92	Myomesin-1-like isoform X2
P348	XP_023124481.1	1275	1789	1796	Pro5	Fast skeletal muscle myosin heavy chain isoform 3
P349	KAA8591518.1	735	7	15	Pro93	AMP deaminase 1 isoform X2
P350	TSK31301.1	343	269	276	Pro7	Glyceraldehyde-3-phosphate dehydrogenase
P351	TSK31301.1	5469	1020	1027	Pro36	Titin
P352	XP_010728431.1	193	4	11	Pro11	Adenylate kinase isoenzyme 1
P353	XP_026208698.1	7374	6627	6636	Pro20	Titin-like
P354	XP_010728431.1	313	292	299	Pro31	Hypothetical protein EPR50_G00074040
P355	ABO31103.1	216	202	209	Pro94	Hypothetical protein EPR50_G00109720
P356	XP_030284270.1	2517	1055	1064	Pro2	Nebulin
