# Supplementary material for: Formation and inhibition mechanism of novel angiotensin I converting enzyme inhibitory peptides from Chouguiyu
Source: Front Nutr. 2022 Jul 22;9:920945. doi: 10.3389/fnut.2022.920945 (PMC9355153; doi:10.3389/fnut.2022.920945)
Supplement: Supplementary file 6 [file Data_Sheet_6.pdf]

Table S3 Characteristics of 30 ACE inhibitory peptides with high abundance from *Chouguiyu*

Number	Isoelectric point	Instability	Water solubility	Toxin	Hydrophobicity (kcal/mol)	Novel	Aliphatic index
P1	5.97	32.83	Good	Non	12.49	Novel	158.75
P2	8.75	-7.67	Poor	Non	9.05	Novel	97.14
P3	6.12	118.50	Good	Non	23.86	Novel	43.33
P4	4.56	4.16	Good	Non	21.91	Novel	43.33
P5	8.63	41.22	Good	Non	14.20	Novel	108.89
P6	8.47	44.00	Poor	Non	10.73	Novel	43.33
P7	3.93	15.43	Good	Non	19.56	Novel	55.71
P8	4.21	8.57	Good	Non	16.44	Novel	55.71
P9	4.21	22.21	Good	Non	15.48	Novel	146.25
P10	5.08	7.47	Poor	Non	14.01	Novel	68.00
P11	3.98	90.77	Good	Non	29.22	Novel	58.00
P12	4.25	70.21	Good	Non	21.96	Novel	72.50
P13	9.75	59.75	Poor	Non	8.31	Novel	85.00
P14	4.20	32.83	Good	Non	15.46	Novel	133.75
P15	8.35	-5.82	Good	Non	15.72	Novel	87.67
P16	6.12	107.65	Good	Non	25.01	Novel	39.00
P17	4.23	-9.72	Good	Non	25.53	Novel	68.00
P18	4.79	58.17	Good	Non	23.05	Novel	58.00
P19	5.21	16.65	Good	Non	22.64	Novel	39.00
P20	5.08	14.61	Poor	Non	11.90	Novel	111.43
P21	8.43	12.82	Good	Non	21.33	Novel	107.00
P22	8.47	15.70	Good	Non	21.33	Novel	107.00
P23	5.99	17.39	Good	Non	19.00	Novel	43.33
P24	8.43	16.33	Good	Non	20.83	Novel	107.78
P25	8.59	62.02	Good	Non	14.71	Novel	136.00
P26	4.37	80.34	Good	Non	16.79	Novel	0.00
P27	6.00	66.78	Poor	Non	11.72	Novel	88.00
P28	10.00	33.26	Good	Non	13.76	Novel	61.25
P29	4.53	100.96	Good	Non	18.87	Novel	43.33
P30	5.08	7.19	Poor	Non	12.86	Novel	75.56

Notes: The instability value <40 means that the structure of peptide is stable.
