# Supplementary material for: Formation and inhibition mechanism of novel angiotensin I converting enzyme inhibitory peptides from Chouguiyu
Source: Front Nutr. 2022 Jul 22;9:920945. doi: 10.3389/fnut.2022.920945 (PMC9355153; doi:10.3389/fnut.2022.920945)
Supplement: Supplementary file 7 [file Data_Sheet_7.PDF]

Table S4 Molecule docking results of ACE and ACE inhibitory peptides

Name	$\Delta E_{docking}$ (Kcal/mol)	$\Delta E_{interaction}$ (Kcal/mol)	$\Delta E_{binding}$ (Kcal/mol)	ΔE_{vdw} (Kcal/mol)	ΔE_{ele} (Kcal/mol)
P1	-136.71	-95.39	-293.44	-29.3538	-305.774
P2	-105.67	-74.95	-193.796	-23.2087	-192.567
P4	-143.95	-108.24	-268.017	-30.3518	-277.428
P7	-124.57	-92.60	-224.219	-23.5153	-227.046
P8	-95.42	-89.40	-145.69	-21.87	-163.12
P9	-113.46	-98.56	-226.977	-32.4958	-244.809
P10	-120.52	-105.68	-326.97	-31.39	-361.35
Captopril	-15.01	-24.44	-15.44	-2.76	-29.23
