# Supplementary material for: Formation and inhibition mechanism of novel angiotensin I converting enzyme inhibitory peptides from Chouguiyu
Source: Front Nutr. 2022 Jul 22;9:920945. doi: 10.3389/fnut.2022.920945 (PMC9355153; doi:10.3389/fnut.2022.920945)
Supplement: Supplementary file 8 [file Data_Sheet_8.PDF]

Table S5 Detailed information of interactions between ACE and ACE inhibitory peptides and captopril

Name	Interaction position	Distance	Category	Type	From	From chemistry	To	To chemistry
P1	Arg124:HH22 - P1:O127	1.91	Hydrogen bond; electrostatic	Salt bridge; attractive charge	Arg124:HH22	H-donor; positive	P1:O127	H-acceptor; negative
	P1:N108 - Glu123:OE1	3.52	Electrostatic	Attractive charge	P1:N108	Positive	Glu123:OE1	Negative
	Lys118:HZ1 - P1:O17	1.84	Hydrogen bond	Conventional hydrogen bond	Lys118:HZ1	H-donor	P1:O17	H-acceptor
	Lys118:HZ2 - P1:N1	2.40	Hydrogen bond	Conventional hydrogen bond	Lys118:HZ2	H-donor	P1:N1	H-acceptor
	Arg124:HH21 - P1:O94	1.95	Hydrogen bond	Conventional hydrogen bond	Arg124:HH21	H-donor	P1:O94	H-acceptor
	Arg522:HH12 - P1:O51	1.85	Hydrogen bond	Conventional hydrogen bond	Arg522:HH12	H-donor	P1:O51	H-acceptor
	Arg522:HH22 - P1:O51	2.31	Hydrogen bond	Conventional hydrogen bond	Arg522:HH22	H-donor	P1:O51	H-acceptor
	P1:H3 - Glu123:OE2	2.93	Hydrogen bond	Conventional hydrogen bond	P1:H3	H-donor	Glu123:OE2	H-acceptor
	P1:H19 - Glu123:OE1	3.09	Hydrogen bond	Conventional hydrogen bond	P1:H19	H-donor	Glu123:OE1	H-acceptor
	P1:H116 - Glu123:OE2	1.96	Hydrogen bond	Conventional hydrogen bond	P1:H116	H-donor	Glu123:OE2	H-acceptor
	P1:H128 - Ser219:O	2.81	Hydrogen bond	Conventional hydrogen bond	P1:H128	H-donor	Ser219:O	H-acceptor
	Lys118:HE1 - P1:N1	2.96	Hydrogen bond	Carbon hydrogen bond	Lys118:HE1	H-donor	P1:N1	H-acceptor
	Ser517:HB2 - P1:O126	2.55	Hydrogen bond	Carbon hydrogen bond	Ser517:HB2	H-donor	P1:O126	H-acceptor
	Pro519:HD2 - P1:O114	2.82	Hydrogen bond	Carbon hydrogen bond	Pro519:HD2	H-donor	P1:O114	H-acceptor
	P1:H5 - Glu123:OE2	2.56	Hydrogen bond	Carbon hydrogen bond	P1:H5	H-donor	Glu123:OE2	H-acceptor

	P1:H120 - Ser516:O	2.60	Hydrogen bond	Carbon hydrogen bond	P1:H120	H-donor	Ser516:O	H-acceptor
	P1:H120 - Ser517:O	2.35	Hydrogen bond	Carbon hydrogen bond	P1:H120	H-donor	Ser517:O	H-acceptor
	P1:C8 - Met223	4.54	Hydrophobic	Alkyl	P1:C8	Alkyl	Met223	Alkyl
	P1:C39 - Met223	5.12	Hydrophobic	Alkyl	P1:C39	Alkyl	Met223	Alkyl
	P1:C39 - Pro407	4.36	Hydrophobic	Alkyl	P1:C39	Alkyl	Pro407	Alkyl
	Phe391 - P1:C65	5.45	Hydrophobic	Pi-alkyl	Phe391	Pi-orbitals	P1:C65	Alkyl
	His410 - P1:C46	4.11	Hydrophobic	Pi-alkyl	His410	Pi-orbitals	P1:C46	Alkyl
P2	Arg124:NH2 - P2:O109	2.95	Electrostatic	Attractive charge	Arg124:NH2	Positive	P2:O109	Negative
	Arg124:HH12 - P2:O108	1.93	Hydrogen bond	Conventional hydrogen bond	Arg124:HH12	H-donor	P2:O108	H-acceptor
	Arg124:HH21 - P2:O31	2.10	Hydrogen bond	Conventional hydrogen bond	Arg124:HH21	H-donor	P2:O31	H-acceptor
	Tyr360:HH - P2:O64	2.95	Hydrogen bond	Conventional hydrogen bond	Tyr360:HH	H-donor	P2:O64	H-acceptor
	P2:H103 - Tyr62:OH	2.07	Hydrogen bond	Conventional hydrogen bond	P2:H103	H-donor	Tyr62:OH	H-acceptor
	P2:H68 - Tyr360:OH	2.46	Hydrogen bond	Carbon hydrogen bond	P2:H68	H-donor	Tyr360:OH	H-acceptor
	Lys118:NZ - P2	4.41	Electrostatic	Pi-cation	Lys118:NZ	Positive	P2	Pi-orbitals
	P2:S58 - Tyr360	6.00	Other	Pi-sulfur	P2:S58	Sulfur	Tyr360	Pi-orbitals
	Phe570 - P2	5.93	Hydrophobic	Pi-pi t-shaped	Phe570	Pi-orbitals	P2	Pi-orbitals
	P2:C38 - Pro519	4.90	Hydrophobic	Alkyl	P2:C38	Alkyl	Pro519	Alkyl
	P2:C42 - Pro519	4.25	Hydrophobic	Alkyl	P2:C42	Alkyl	Pro519	Alkyl
	Trp59 - P2:C71	3.56	Hydrophobic	Pi-alkyl	Trp59	Pi-orbitals	P2:C71	Alkyl
	Trp59 - P2:C75	4.59	Hydrophobic	Pi-alkyl	Trp59	Pi-orbitals	P2:C75	Alkyl
	P2 - Met223	4.96	Hydrophobic	Pi-alkyl	P2	Pi-orbitals	Met223	Alkyl
P4	Arg124:HH12 - P4:O14	1.96	Hydrogen bond; electrostatic	Salt bridge; attractive charge	Arg124:HH12	H-donor; positive	P4:O14	H-acceptor; negative
	Arg522:HH12 - P4:O106	2.34	Hydrogen bond; electrostatic	Salt bridge; attractive charge	Arg522:HH12	H-donor;positive	P4:O106	H-acceptor; negative

	Arg522:HH22 - P4:O106	1.89	Hydrogen bond; electrostatic	Salt bridge; attractive charge	Arg522:HH22	H-donor; positive	P4:O106	H-acceptor; negative
	Arg124:NH2 - P4:O94	2.92	Electrostatic	Attractive charge	Arg124:NH2	Positive	P4:O94	Negative
	Tyr62:HH - P4:O13	2.09	Hydrogen bond	Conventional hydrogen bond	Tyr62:HH	H-donor	P4:O13	H-acceptor
	Asn66:HD22 - P4:O16	2.49	Hydrogen bond	Conventional hydrogen bond	Asn66:HD22	H-donor	P4:O16	H-acceptor
	Asn85:HD22 - P4:O13	2.46	Hydrogen bond	Conventional hydrogen bond	Asn85:HD22	H-donor	P4:O13	H-acceptor
	Arg124:HN - P4:O93	2.44	Hydrogen bond	Conventional hydrogen bond	Arg124:HN	H-donor	P4:O93	H-acceptor
	Arg124:HE - P4:O93	1.98	Hydrogen bond	Conventional hydrogen bond	Arg124:HE	H-donor	P4:O93	H-acceptor
	P4:H147 - Met223:O	2.90	Hydrogen bond	Conventional hydrogen bond	P4:H147	H-donor	Met223:O	H-acceptor
	P4:H148 - Gly404:O	2.93	Hydrogen bond	Conventional hydrogen bond	P4:H148	H-donor	Gly404:O	H-acceptor
	Pro407:HD2 - P4:N146	2.35	Hydrogen bond	Carbon hydrogen bond	Pro407:HD2	H-donor	P4:N146	H-acceptor
	Pro519:HD1 - P4:O105	2.48	Hydrogen bond	Carbon hydrogen bond	Pro519:HD1	H-donor	P4:O105	H-acceptor
	Pro519:HD2 - P4:O105	2.61	Hydrogen bond	Carbon hydrogen bond	Pro519:HD2	H-donor	P4:O105	H-acceptor
	P4:H5 - Tyr62:OH	2.39	Hydrogen bond	Carbon hydrogen bond	P4:H5	H-donor	Tyr62:OH	H-acceptor
	P4:H112 - Glu123:OE2	2.74	Hydrogen bond	Carbon hydrogen bond	P4:H112	H-donor	Glu123:OE 2	H-acceptor
	P4:H144 - Gly404:O	2.64	Hydrogen bond	Carbon hydrogen bond	P4:H144	H-donor	Gly404:O	H-acceptor
	P4:H145 - Gly404:O	2.90	Hydrogen bond	Carbon hydrogen bond	P4:H145	H-donor	Gly404:O	H-acceptor
	Met223:SD - P4:O129	3.08	Other	Sulfur-x	Met223:SD	Sulfur	P4:O129	O,n,s
	P4:C30 - Val518	4.94	Hydrophobic	Alkyl	P4:C30	Alkyl	Val518	Alkyl
	Phe512 - P4:C30	5.09	Hydrophobic	Pi-alkyl	Phe512	Pi-orbitals	P4:C30	Alkyl
P7	Lys118:HZ2 - P7:O11	1.98	Hydrogen bond; electrostatic	Salt bridge; attractive charge	Lys118:HZ2	H-donor; positive	P7:O11	H-acceptor; negative

Arg124:NH1 - P7:O115	3.99	Electrostatic	Attractive charge	Arg124:NH1	Positive	P7:O115	Negative
Arg522:NH2 - P7:O43	3.54	Electrostatic	Attractive charge	Arg522:NH2	Positive	P7:O43	Negative
Arg522:NH2 - P7:O55	5.18	Electrostatic	Attractive charge	Arg522:NH2	Positive	P7:O55	Negative
Lys118:HZ1 - P7:O13	1.87	Hydrogen bond	Conventional hydrogen bond	Lys118:HZ1	H-donor	P7:O13	H-acceptor
Arg124:HE - P7:O88	2.27	Hydrogen bond	Conventional hydrogen bond	Arg124:HE	H-donor	P7:O88	H-acceptor
Arg124:HH12 - P7:O114	2.01	Hydrogen bond	Conventional hydrogen bond	Arg124:HH12	H-donor	P7:O114	H-acceptor
Arg124:HH21 - P7:O88	1.82	Hydrogen bond	Conventional hydrogen bond	Arg124:HH21	H-donor	P7:O88	H-acceptor
Arg124:HH22 - P7:O114	2.07	Hydrogen bond	Conventional hydrogen bond	Arg124:HH22	H-donor	P7:O114	H-acceptor
Trp220:HE1 - P7:O54	2.88	Hydrogen bond	Conventional hydrogen bond	Trp220:HE1	H-donor	P7:O54	H-acceptor
Arg522:HH12 - P7:O42	2.25	Hydrogen bond	Conventional hydrogen bond	Arg522:HH12	H-donor	P7:O42	H-acceptor
Arg522:HH22 - P7:O42	2.05	Hydrogen bond	Conventional hydrogen bond	Arg522:HH22	H-donor	P7:O42	H-acceptor
P7:H2 - Glu123:OE1	2.54	Hydrogen bond	Conventional hydrogen bond	P7:H2	H-donor	Glu123:OE 1	H-acceptor
P7:H35 - Glu403:OE2	2.29	Hydrogen bond	Conventional hydrogen bond	P7:H35	H-donor	Glu403:OE 2	H-acceptor
P7:H78 - Ser517:O	2.38	Hydrogen bond	Conventional hydrogen bond	P7:H78	H-donor	Ser517:O	H-acceptor
P7:H91 - Glu123:OE1	2.58	Hydrogen bond	Conventional hydrogen bond	P7:H91	H-donor	Glu123:OE 1	H-acceptor
P7:H111 - Asn70:OD1	2.20	Hydrogen bond	Conventional hydrogen bond	P7:H111	H-donor	Asn70:OD1	H-acceptor
Ser517:HB2 - P7:O93	2.29	Hydrogen bond	Carbon hydrogen bond	Ser517:HB2	H-donor	P7:O93	H-acceptor
Pro519:HD2 - P7:O55	2.54	Hydrogen bond	Carbon hydrogen bond	Pro519:HD2	H-donor	P7:O55	H-acceptor

	P7:H5 - Glu123:OE1	2.45	Hydrogen bond	Carbon hydrogen bond	P7:H5	H-donor	Glu123:OE1	H-acceptor
	P7:H109 - Glu143:OE1	2.57	Hydrogen bond	Carbon hydrogen bond	P7:H109	H-donor	Glu143:OE1	H-acceptor
	P7:O55 - Trp220	3.78	Electrostatic	Pi-anion	P7:O55	Negative	Trp220	Pi-orbitals
	Trp59 - P7	5.47	Hydrophobic	Pi-pi t-shaped	Trp59	Pi-orbitals	P7	Pi-orbitals
	P7:C64 - Val518	4.63	Hydrophobic	Alkyl	P7:C64	Alkyl	Val518	Alkyl
P8	Arg124:NH2 - P8:O37	5.03	Electrostatic	Attractive charge	Arg124:NH2	Positive	P8:O37	Negative
	Arg522:NH1 - P8:O49	3.18	Electrostatic	Attractive charge	Arg522:NH1	Positive	P8:O49	Negative
	Asn66:HD21 - P8:O86	2.19	Hydrogen bond	Conventional hydrogen bond	Asn66:HD21	H-donor	P8:O86	H-acceptor
	Trp220:HE1 - P8:O36	2.30	Hydrogen bond	Conventional hydrogen bond	Trp220:HE1	H-donor	P8:O36	H-acceptor
	Trp220:HE1 - P8:O39	2.88	Hydrogen bond	Conventional hydrogen bond	Trp220:HE1	H-donor	P8:O39	H-acceptor
	Tyr360:HH - P8:O106	2.64	Hydrogen bond	Conventional hydrogen bond	Tyr360:HH	H-donor	P8:O106	H-acceptor
	Arg522:HH12 - P8:O48	1.89	Hydrogen bond	Conventional hydrogen bond	Arg522:HH12	H-donor	P8:O48	H-acceptor
	Arg522:HH22 - P8:O48	2.16	Hydrogen bond	Conventional hydrogen bond	Arg522:HH22	H-donor	P8:O48	H-acceptor
	P8:H2 - Glu123:OE2	2.14	Hydrogen bond	Conventional hydrogen bond	P8:H2	H-donor	Glu123:OE2	H-acceptor
	P8:H22 - Glu123:OE2	2.22	Hydrogen bond	Conventional hydrogen bond	P8:H22	H-donor	Glu123:OE2	H-acceptor
	Pro519:HD1 - P8:O49	2.59	Hydrogen bond	Carbon hydrogen bond	Pro519:HD1	H-donor	P8:O49	H-acceptor
	P8:H55 - Ser517:O	2.70	Hydrogen bond	Carbon hydrogen bond	P8:H55	H-donor	Ser517:O	H-acceptor
	P8:H83 - Trp59	2.76	Hydrogen bond	Pi-donor hydrogen bond	P8:H83	H-donor	Trp59	Pi-orbitals
	P8:H83 - Trp59	3.01	Hydrogen bond	Pi-donor hydrogen bond	P8:H83	H-donor	Trp59	Pi-orbitals
	Trp857 - P8	5.30	Hydrophobic	Pi-pi t-shaped	Trp857	Pi-orbitals	P8	Pi-orbitals
	Trp857 - P8	5.31	Hydrophobic	Pi-pi t-shaped	Trp857	Pi-orbitals	P8	Pi-orbitals
	Val518 - P8	4.93	Hydrophobic	Alkyl	Val518	Alkyl	P8	Alkyl

	P8:C15 - Met223	4.25	Hydrophobic	Alkyl	P8:C15	Alkyl	Met223	Alkyl
	Phe570 - P8:C15	4.65	Hydrophobic	Pi-alkyl	Phe570	Pi-orbitals	P8:C15	Alkyl
P9	Arg124:HH21 - P9:O56	2.01	Hydrogen bond; electrostatic	Salt bridge; attractive charge	Arg124:HH21	H-donor; positive	P9:O56	H-acceptor; negative
	Lys368:NZ - P9:O132	4.19	Electrostatic	Attractive charge	Lys368:NZ	Positive	P9:O132	Negative
	Arg522:NH2 - P9:O44	4.90	Electrostatic	Attractive charge	Arg522:NH2	Positive	P9:O44	Negative
	Asn66:HD22 - P9:O93	2.58	Hydrogen bond	Conventional hydrogen bond	Asn66:HD22	H-donor	P9:O93	H-acceptor
	Lys118:HZ1 - P9:N1	2.07	Hydrogen bond	Conventional hydrogen bond	Lys118:HZ1	H-donor	P9:N1	H-acceptor
	Lys118:HZ2 - P9:O20	2.30	Hydrogen bond	Conventional hydrogen bond	Lys118:HZ2	H-donor	P9:O20	H-acceptor
	Arg124:HN - P9:O55	2.24	Hydrogen bond	Conventional hydrogen bond	Arg124:HN	H-donor	P9:O55	H-acceptor
	Arg124:HH22 - P9:O72	2.52	Hydrogen bond	Conventional hydrogen bond	Arg124:HH22	H-donor	P9:O72	H-acceptor
	Arg522:HH12 - P9:O43	2.46	Hydrogen bond	Conventional hydrogen bond	Arg522:HH12	H-donor	P9:O43	H-acceptor
	Arg522:HH22 - P9:O43	1.89	Hydrogen bond	Conventional hydrogen bond	Arg522:HH22	H-donor	P9:O43	H-acceptor
	P9:H31 - ASP121:OD2	2.91	Hydrogen bond	Conventional hydrogen bond	P9:H31	H-donor	ASP121:OD2	H-acceptor
	P9:H32 - Glu123:OE2	2.44	Hydrogen bond	Conventional hydrogen bond	P9:H32	H-donor	Glu123:OE2	H-acceptor
	P9:H48 - Glu123:OE1	2.34	Hydrogen bond	Conventional hydrogen bond	P9:H48	H-donor	Glu123:OE1	H-acceptor
	Lys118:HE1 - P9:O20	3.07	Hydrogen bond	Carbon hydrogen bond	Lys118:HE1	H-donor	P9:O20	H-acceptor
	Met223:HA - P9:O29	2.84	Hydrogen bond	Carbon hydrogen bond	Met223:HA	H-donor	P9:O29	H-acceptor
	P9:H5 - Glu403:OE2	3.09	Hydrogen bond	Carbon hydrogen bond	P9:H5	H-donor	Glu403:OE2	H-acceptor

	P9:H24 - Glu123:OE2	2.50	Hydrogen bond	Carbon hydrogen bond	P9:H24	H-donor	Glu123:OE2	H-acceptor
	P9:H50 - Glu123:OE1	2.55	Hydrogen bond	Carbon hydrogen bond	P9:H50	H-donor	Glu123:OE1	H-acceptor
	Arg124 - P9	4.91	Hydrophobic	Alkyl	Arg124	Alkyl	P9	Alkyl
	Trp59 - P9:C8	4.87	Hydrophobic	Pi-alkyl	Trp59	Pi-orbitals	P9:C8	Alkyl
P10	Arg124:HH21 - P10:O142	2.83	Hydrogen bond; electrostatic	Salt bridge; attractive charge	Arg124:HH21	H-donor; positive	P10:O142	H-acceptor; negative
	Arg124:HH21 - P10:O141	2.85	Hydrogen bond	Conventional hydrogen bond	Arg124:HH21	H-donor	P10:O141	H-acceptor
	Gly404:HN - P10:O34	2.14	Hydrogen bond	Conventional hydrogen bond	Gly404:HN	H-donor	P10:O34	H-acceptor
	Arg522:HH22 - P10:O24	2.74	Hydrogen bond	Conventional hydrogen bond	Arg522:HH22	H-donor	P10:O24	H-acceptor
	P10:H3 - Glu403:OE2	2.03	Hydrogen bond	Conventional hydrogen bond	P10:H3	H-donor	Glu403:OE2	H-acceptor
	P10:H10 - Glu403:OE2	3.03	Hydrogen bond	Conventional hydrogen bond	P10:H10	H-donor	Glu403:OE2	H-acceptor
	P10:H47 - Arg402:O	2.21	Hydrogen bond	Conventional hydrogen bond	P10:H47	H-donor	Arg402:O	H-acceptor
	P10:H66 - Tyr360:OH	2.64	Hydrogen bond	Conventional hydrogen bond	P10:H66	H-donor	Tyr360:OH	H-acceptor
	P10:H83 - Tyr62:OH	2.15	Hydrogen bond	Conventional hydrogen bond	P10:H83	H-donor	Tyr62:OH	H-acceptor
	Pro407:HA - P10:O33	2.54	Hydrogen bond	Carbon hydrogen bond	Pro407:HA	H-donor	P10:O33	H-acceptor
	His410:HD2 - P10:O34	2.70	Hydrogen bond	Carbon hydrogen bond	His410:HD2	H-donor	P10:O34	H-acceptor
	P10:H5 - Glu123:OE1	2.75	Hydrogen bond	Carbon hydrogen bond	P10:H5	H-donor	Glu123:OE1	H-acceptor
	Arg124:NH2 - P10	3.93	Electrostatic	Pi-cation	Arg124:NH2	Positive	P10	Pi-orbitals
	P10:H21 - Phe570	2.65	Hydrophobic	Pi-sigma	P10:H21	C-h	Phe570	Pi-orbitals
	P10:C15 - Met223	4.81	Hydrophobic	Alkyl	P10:C15	Alkyl	Met223	Alkyl
	P10:C15 - Pro407	3.98	Hydrophobic	Alkyl	P10:C15	Alkyl	Pro407	Alkyl
	P10:C129 - LEU139	4.39	Hydrophobic	Alkyl	P10:C129	Alkyl	LEU139	Alkyl
	Trp357 - P10	4.69	Hydrophobic	Pi-alkyl	Trp357	Pi-orbitals	P10	Alkyl

	Phe570 - P10:C15	4.62	Hydrophobic	Pi-alkyl	Phe570	Pi-orbitals	P10:C15	Alkyl
Captopril	Arg124:HE - captopril:O6	2.07	Hydrogen bond	Conventional hydrogen bond	Arg124:HE	H-donor	captopril:O6	H-acceptor
	Arg124:HH21 - captopril:O6	2.08	Hydrogen bond	Conventional hydrogen bond	Arg124:HH21	H-donor	captopril:O6	H-acceptor
	Trp220:HE1 - captopril:O14	2.54	Hydrogen bond	Conventional hydrogen bond	Trp220:HE1	H-donor	captopril:O14	H-acceptor
	Ser517:HG - captopril:O14	3.00	Hydrogen bond	Conventional hydrogen bond	Ser517:HG	H-donor	captopril:O14	H-acceptor
	captopril:H29 - Tyr135:OH	1.96	Hydrogen bond	Conventional hydrogen bond	captopril:H29	H-donor	Tyr135:OH	H-acceptor
	captopril:H22 - Glu123:OE1	2.45	Hydrogen bond	Carbon hydrogen bond	captopril:H22	H-donor	Glu123:OE1	H-acceptor
	Trp220 - captopril	5.19	Hydrophobic	Pi-alkyl	Trp220	Pi-orbitals	captopril	Alkyl
