# Supplementary material for: Formation and inhibition mechanism of novel angiotensin I converting enzyme inhibitory peptides from Chouguiyu
Source: Front Nutr. 2022 Jul 22;9:920945. doi: 10.3389/fnut.2022.920945 (PMC9355153; doi:10.3389/fnut.2022.920945)
Supplement: Supplementary file 9 [file Data_Sheet_9.PDF]

Table S6 Cleavage enzymes of P1, P2, P7, P8, P9, and P10 and their tentative microbial source with COG annotation

Peptides	Predicted proteases	Microbial genus with significantly positive correlation and COG
P1: F-V E I I N A R A	Serine endopeptidases (EC 3.4.21.1; EC; 3.4.21.20; EC 3.4.21.3; EC 3.4.21.39; EC 3.4.21.62; EC 3.4.21.67; EC 3.4.21.71)	*Aeromonas* (r=0.69): OG1770, COG0466, COG1219, COG0265, COG0681; *Bacillus* (r=0.62): COG1506, COG0542, COG1404, COG1505, ENOG410XP7P
	Cysteine endopeptidases (EC 3.4.22.2; EC 3.4.22.3; EC 3.4.22.32)	*Aeromonas* (r=0.69): COG3672
	Aspartic endopeptidases (EC 3.4.23.1)	*Aeromonas* (r=0.69): COG0616, COG3577
	Metalloendopeptidases (EC 3.4.24.27; EC 3.4.24.30)	*Aeromonas* (r=0.69): COG0826, COG0750, COG0501, COG4942, COG1164; *Bacillus* (r=0.62): COG5504, COG3340, COG0465, COG3740, COG0501
P2: E-F A V M V K G	Serine endopeptidases (EC 3.4.21.19; EC 3.4.21.2)	*Escherichia* (r=0.84): COG1770, ENOG410XP7P, COG0542, COG0265, ENOG4111MMN; *Bacillus* (r=0.82): COG1506, COG0542, COG1404, COG1505, ENOG410XP7P
	Aspartic endopeptidases (EC 3.4.23.1)	*Escherichia* (r=0.84): COG0616
	Metalloendopeptidases (EC 3.4.24.27; EC 3.4.24.30)	*Escherichia* (r=0.84): COG0739, ENOG410XZ09, COG0465, COG0339; *Bacillus* (r=0.82): COG5504, COG3340, COG0465, COG3740, COG0501
P4: Y-E I T W S D D K K	Serine endopeptidases (EC 3.4.21.1; EC 3.4.21.2; EC 3.4.21.20; EC 3.4.21.3; EC 3.4.21.36; EC 3.4.21.39; EC 3.4.21.67; EC3.4.21.62)	*Escherichia* (r=0.63): COG1770, NOG410XP7P, COG0542, COG0265, ENOG4111MMN; *Bacillus* (r=0.60): COG1506, COG0542, COG1404, COG1505, ENOG410XP7P
	Aspartic endopeptidases (EC 3.4.23.1)	*Escherichia* (r=0.63): COG0616
P7: V-D F D D I Q K-K	Cleavage at N-terminal	
	Serine endopeptidases (EC 3.4.21.36; EC 3.4.21.37; EC 3.4.21.67; EC 3.4.21.96)	*Clostridium* (r=0.90): COG0542, COG1404, COG0793, COG0265, COG1067; *Lactococcus* (r=0.86): COG0265, COG0542, COG0681, COG1219, COG1404; *Peptostreptococcus* (r=0.84): COG0466, COG1219, COG0793, COG0542, COG0265;

		Citrobacter (r=0.84): COG0265, COG0793, COG1067, COG1770, COG0542;<br>*Streptococcus* (r=0.84): COG0542, COG1404, COG0681, COG0265, COG1219;<br>*Vagococcus* (r=0.78): COG0542, COG0681, COG0793, COG1506, COG0265;<br>*Enterococcus* (r=0.78): COG0542, COG0681, ENOG4111WMB, COG1506, COG0793;<br>*Fusobacterium* (r=0.76): COG1219, COG0681, COG0793, COG0542, COG0466;<br>*Morganella* (r=0.71): COG0466, COG0542, COG1770, COG1219, COG0681;<br>*Hafnia* (r=0.71): COG0542, COG0681, COG0265, COG1770, COG0793;<br>*Carnobacterium* (r=0.70): COG0542, COG0265, COG0542, COG0793, COG1219;<br>*Psychrobacter* (r=0.64): COG0466, COG0542, COG1219, COG1067, COG0265;<br>*Vibrio* (r=0.60): COG0681, COG1067, COG0466, COG0265, COG2802
	Cysteine endopeptidases (EC 3.4.22.32)	*Citrobacter* (r=0.84): COG3672;<br>*Psychrobacter* (r=0.64): ENOG4111IPD;<br>*Vibrio* (r=0.60): COG3672
	Cleavage at C-terminal	
	Serine endopeptidases (EC 3.4.21.4; EC 3.4.21.7; EC 3.4.21.83)	*Clostridium* (r=0.90): COG0542, COG1404, COG0793, COG0265, COG1067;<br>*Lactococcus* (r=0.86): COG0265, COG0542, COG0681, COG1219, COG1404;<br>*Peptostreptococcus* (r=0.84): COG0466, COG1219, COG0793, COG0542, COG0265;<br>*Citrobacter* (r=0.84): COG0265, COG0793, COG1067, COG1770, COG0542;<br>*Streptococcus* (r=0.84): COG0542, COG1404, COG0681, COG0265, COG1219;<br>*Vagococcus* (r=0.78): COG0542, COG0681, COG0793, COG1506, COG0265;<br>*Enterococcus* (r=0.78): COG0542, COG0681, ENOG4111WMB, COG1506, COG0793;<br>*Fusobacterium* (r=0.76): COG1219, COG0681, COG0793, COG0542, COG0466;<br>*Morganella* (r=0.71): COG0466, COG0542, COG1770, COG1219, COG0681;<br>*Hafnia* (r=0.71): COG0542, COG0681, COG0265, COG1770, COG0793;<br>*Carnobacterium* (r=0.70): COG0542, COG0265, COG0542, COG0793, COG1219;<br>*Psychrobacter* (r=0.64): COG0466, COG0542, COG1219, COG1067, COG0265;<br>*Vibrio* (r=0.60): COG0681, COG1067, COG0466, COG0265, COG2802
	Cysteine endopeptidases (EC 3.4.22.3; EC 3.4.22.53)	*Citrobacter* (r=0.84): COG3672;<br>*Psychrobacter* (r=0.64): ENOG4111IPD;<br>*Vibrio* (r=0.60): COG3672
	Aspartic endopeptidases	*Lactococcus* (r=0.86): ENOG41129X1;

	(EC 3.4.23.1)	*Citrobacter* (r=0.84): COG0616;<br>*Vagococcus* (r=0.78): COG0616, ENOG41129X1;<br>*Enterococcus* (r=0.78): COG0616;<br>*Fusobacterium* (r=0.76): COG0616;<br>*Morganella* (r=0.71): COG0616, ENOG410Y2Y1, ENOG41126WC;<br>*Hafnia* (r=0.71)： COG0616, ENOG41126WC;<br>*Carnobacterium* (r=0.70): COG0616;<br>*Psychrobacter* (r=0.64): COG0616;<br>*Vibrio* (r=0.60): COG0616, ENOG410Y2Y1
P8: I-I G D D P K F-R	Cleavage at N-terminal	
	Serine endopeptidases (EC 3.4.21.36; EC 3.4.21.37; EC 3.4.21.67)	*Clostridium* (r=0.90): COG0542, COG1404, COG0793, COG0265, COG1067;<br>*Citrobacter* (r=0.86): COG0265, COG0793, COG1067, COG1770, COG0542;<br>*Lactococcus* (r=0.82): COG0265, COG0542, COG0681, COG1219, COG1404;<br>*Peptostreptococcus* (r=0.80): COG0466, COG1219, COG0793, COG0542, COG0265;<br>*Streptococcus* (r=0.80): COG0542, COG1404, COG0681, COG0265, COG1219;<br>*Fusobacterium* (r=0.79): COG1219, COG0681, COG0793, COG0542, COG0466;<br>*Morganella* (r=0.74): COG0466, COG0542, COG1770, COG1219, COG0681;<br>*Hafnia* (r=0.74): COG0542, COG0681, COG0265, COG1770, COG0793;<br>*Vagococcus* (r=0.73): COG0542, COG0681, COG0793, COG1506, COG0265;<br>*Enterococcus* (r=0.72): COG0542, COG0681, ENOG4111WMB, COG1506, COG0793;<br>*Vibrio* (r=0.64): COG0681, COG1067, COG0466, COG0265, COG2802;<br>*Carnobacterium* (r=0.64): COG0265, COG0542, COG0793, COG1219, COG1404;<br>*Psychrilyobacter* (r=0.63): COG0542, COG0466, COG0793;<br>*Pseudoalteromonas* (r=0.61): COG0265, COG0681, COG1506, COG0466, COG1404
	Aspartic endopeptidases (EC 3.4.23.1)	*Citrobacter* (r=0.86): COG0616;<br>*Lactococcus* (r=0.82): ENOG41129X1;<br>*Fusobacterium* (r=0.79): COG0616;<br>*Morganella* (r=0.74): COG0616, ENOG410Y2Y1, ENOG41126WC, ENOG41126WC;<br>*Hafnia* (r=0.74): COG0616, ENOG41126WC, ENOG41126WC;<br>*Vagococcus* (r=0.73): COG0616, ENOG41129X1;

		Enterococcus (r=0.72): COG0616;<br>*Vibrio* (r=0.64): COG0616, ENOG410Y2Y1;<br>*Carnobacterium* (r=0.64): COG0616;<br>*Psychrilyobacter* (r=0.63): COG0616, COG4571;<br>*Pseudoalteromonas* (r=0.61): COG0616, ENOG410Y2Y1
	Metalloendopeptidases (EC 3.4.24.27; EC 3.4.24.30)	*Clostridium* (r=0.90): COG0465, COG5504, COG0006, ENOG410XQ69;<br>*Citrobacter* (r=0.86): COG0339, COG4942, COG0612, COG1214, COG0750;<br>*Lactococcus* (r=0.82): COG0465, COG0006, COG0612, COG1164, COG0826;<br>*Peptostreptococcus* (r=0.80): COG1164, COG0612, COG0465, COG0826, COG0006;<br>*Streptococcus* (r=0.80): COG0465, COG0612, COG0006, COG0826, COG1214;<br>*Fusobacterium* (r=0.79): COG4942, COG0826, COG0465, COG0006, COG0612;<br>*Morganella* (r=0.74): COG0826, COG3340, COG0739, ENOG410XRMR, COG2321;<br>*Hafnia* (r=0.74): COG0739, COG2234, COG0750, COG0612, COG0339;<br>*Vagococcus* (r=0.73): COG0739, COG0826, ENOG4110293, COG0006, COG0465;<br>*Enterococcus* (r=0.72): COG2738, COG0739, COG0612, COG3212, COG0465;<br>*Vibrio* (r=0.64): COG4942, COG1164, COG0465, ENOG41121TG, COG0739;<br>*Carnobacterium* (r=0.64): COG1164, COG0465, COG0612, COG1214, COG0750;<br>*Psychrilyobacter* (r=0.63): COG0612, COG1026, COG0006, COG0826, COG1164;<br>*Pseudoalteromonas* (r=0.61): COG0006, COG0750, ENOG410XSFU, COG0465, COG0739
	Cleavage at C-terminal	
	Serine endopeptidases (EC 3.4.21.1; EC 3.4.21.20; EC 3.4.21.3; EC 3.4.21.39; EC 3.4.21.62; EC 3.4.21.71; EC 3.4.21.67)	*Clostridium* (r=0.90): COG0542, COG1404, COG0793, COG0265, COG1067;<br>*Citrobacter* (r=0.86): COG0265, COG0793, COG1067, COG1770, COG0542;<br>*Lactococcus* (r=0.82): COG0265, COG0542, COG0681, COG1219, COG1404;<br>*Peptostreptococcus* (r=0.80): COG0466, COG1219, COG0793, COG0542, COG0265;<br>*Streptococcus* (r=0.80): COG0542, COG1404, COG0681, COG0265, COG1219;<br>*Fusobacterium* (r=0.79): COG1219, COG0681, COG0793, COG0542, COG0466;<br>*Morganella* (r=0.74): COG0466, COG0542, COG1770, COG1219, COG0681;<br>*Hafnia* (r=0.74): COG0542, COG0681, COG0265, COG1770, COG0793;<br>*Vagococcus* (r=0.73): COG0542, COG0681, COG0793, COG1506, COG0265;

		Enterococcus (r=0.72): COG0542, COG0681, ENOG4111WMB, COG1506, COG0793; *Vibrio* (r=0.64): COG0681, COG1067, COG0466, COG0265, COG2802;<br>*Carnobacterium* (r=0.64): COG0265, COG0542, COG0793, COG1219, COG1404;<br>*Psychrilyobacter* (r=0.63): COG0542, COG0466, COG0793;<br>*Pseudoalteromonas* (r=0.61): COG0265, COG0681, COG1506, COG0466, COG1404
	Cysteine endopeptidases (EC 3.4.22.2; EC 3.4.22.3; EC 3.4.22.32)	*Citrobacter* (r=0.86): COG3672;<br>*Vibrio* (r=0.64): COG3672;<br>*Pseudoalteromonas* (r=0.61): COG3672
	Aspartic endopeptidases (EC 3.4.23.1)	*Citrobacter* (r=0.86): COG0616;<br>*Lactococcus* (r=0.82): ENOG41129X1;<br>*Fusobacterium* (r=0.79): COG0616;<br>*Morganella* (r=0.74): COG0616, ENOG410Y2Y1, ENOG41126WC, ENOG41126WC;<br>*Hafnia* (r=0.74): COG0616, ENOG41126WC, ENOG41126WC;<br>*Vagococcus* (r=0.73): COG0616, ENOG41129X1;<br>*Enterococcus* (r=0.72): COG0616;<br>*Vibrio* (r=0.64): COG0616, ENOG410Y2Y1;<br>*Carnobacterium* (r=0.64): COG0616;<br>*Psychrilyobacter* (r=0.63): COG0616, COG4571;<br>*Pseudoalteromonas* (r=0.61): COG0616, ENOG410Y2Y1
P9:S-I N D D P K I L-H	Cleavage at N-terminal	
	Serine endopeptidases (EC 3.4.21.36; EC 3.4.21.37; EC 3.4.21.62)	*Lactococcus* (r=0.89): COG0265, COG0542, COG0681, COG1219, COG1404;<br>*Peptostreptococcus* (r=0.88): COG0466, COG1219, COG0793, COG0542, COG0265;<br>*Streptococcus* (r=0.88): COG0542, COG1404, COG0681, COG0265, COG1219;<br>*Clostridium* (r=0.87): COG0542, COG1404, COG0793, COG0265, COG1067;<br>*Vagococcus* (r=0.83): COG0542, COG0681, COG0793, COG1506, COG0265;<br>*Enterococcus* (r=0.83): COG0542, COG0681, ENOG4111WMB, COG1506, COG0793;<br>*Citrobacter* (r=0.79): COG0265, COG0793, COG1067, COG1770, COG0542;<br>*Carnobacterium* (r=0.77): COG0265, COG0542, COG0793, COG1219, COG1404;<br>*Psychrobacter* (r=0.71): COG0466, COG0542, COG1219, COG1067, COG0265;<br>*Fusobacterium* (r=0.69): COG1219, COG0681, COG0793, COG0542, COG0466;

	Morganella (r=0.63): COG0466, COG0542, COG1770, COG1219, COG0681; *Hafnia* (r=0.63): COG0542, COG0681, COG0265, COG1770, COG0793
Cysteine endopeptidases (EC 3.4.22.3; EC 3.4.22.32)	*Citrobacter* (r=0.79): COG3672; *Psychrobacter* (r=0.71): ENOG4111IPD;
Aspartic endopeptidases (EC 3.4.23.1)	*Lactococcus* (r=0.89): ENOG41129X1; *Vagococcus* (r=0.83): COG0616, ENOG41129X1; *Enterococcus* (r=0.83): COG0616; *Citrobacter* (r=0.79): COG0616; *Carnobacterium* (r=0.77): COG0616; *Psychrobacter* (r=0.71): COG0616; *Fusobacterium* (r=0.69): COG0616; *Morganella* (r=0.63): COG0616, ENOG410Y2Y1, ENOG41126WC; *Hafnia* (r=0.63): COG0616, ENOG41126WC
Metalloendopeptidases (EC 3.4.24.27; EC 3.4.24.30)	*Lactococcus* (r=0.89): COG0465, COG0006, COG0612, COG1164, COG0826; *Peptostreptococcus* (r=0.88): COG1164, COG0612, COG0465, COG0826, COG0006; *Streptococcus* (r=0.88): COG0465, COG0612, COG0006, COG0826, COG1214; *Clostridium* (r=0.87): COG0465, COG1214, COG5504, COG0006, ENOG410XQ69; *Vagococcus* (r=0.83): COG0739, COG0826, ENOG4110293, COG0006, COG0465; *Enterococcus* (r=0.83): COG2738, COG0739, COG0612, COG0465, ENOG4112DC9; *Citrobacter* (r=0.79): COG0339, COG4942, COG0612, COG1214, COG0750; *Carnobacterium* (r=0.77): COG1164, COG0465, COG0612, COG1214, COG0750; *Psychrobacter* (r=0.71): COG0339, COG0465, COG4783, COG1026, COG0739; *Fusobacterium* (r=0.69): COG4942, COG0826, COG0465, COG0006, COG0612; *Morganella* (r=0.63): COG0826, COG3340, COG0739, ENOG410XRMR, COG2321; *Hafnia* (r=0.63): COG0739, COG2234, COG0750, COG0612, COG0339
Cleavage at C-terminal	
Serine endopeptidases (EC 3.4.21.1; EC 3.4.21.2; EC 3.4.21.20; EC 3.4.21.3; EC 3.4.21.39; EC 3.4.21.67; EC 3.4.21.71; EC 3.4.21.96; EC	*Lactococcus* (r=0.89): COG0265, COG0542, COG0681, COG1219, COG1404; *Peptostreptococcus* (r=0.88): COG0466, COG1219, COG0793, COG0542, COG0265; *Streptococcus* (r=0.88): COG0542, COG1404, COG0681, COG0265, COG1219; *Clostridium* (r=0.87): COG0542, COG1404, COG0793, COG0265, COG1067;

	3.4.21.36; EC 3.4.21.37; EC 3.4.21.62)	*Vagococcus* (r=0.83): COG0542, COG0681, COG0793, COG1506, COG0265; *Enterococcus* (r=0.83): COG0542, COG0681, ENOG4111WMB, COG1506, COG0793; *Citrobacter* (r=0.79): COG0265, COG0793, COG1067, COG1770, COG0542; *Carnobacterium* (r=0.77): COG0265, COG0542, COG0793, COG1219, COG1404; *Psychrobacter* (r=0.71): COG0466, COG0542, COG1219, COG1067, COG0265; *Fusobacterium* (r=0.69): COG1219, COG0681, COG0793, COG0542, COG0466; *Morganella* (r=0.63): COG0466, COG0542, COG1770, COG1219, COG0681; *Hafnia* (r=0.63): COG0542, COG0681, COG0265, COG1770, COG0793
	Cysteine endopeptidases (EC 3.4.22.2; EC 3.4.22.53; EC 3.4.22.3; EC 3.4.22.32)	*Citrobacter* (r=0.79): COG3672; *Psychrobacter* (r=0.71): ENOG4111IPD;
	Aspartic endopeptidases (EC 3.4.23.1)	*Lactococcus* (r=0.89): ENOG41129X1; *Vagococcus* (r=0.83): COG0616, ENOG41129X1; *Enterococcus* (r=0.83): COG0616; *Citrobacter* (r=0.79): COG0616; *Carnobacterium* (r=0.77): COG0616; *Psychrobacter* (r=0.71): COG0616; *Fusobacterium* (r=0.69): COG0616; *Morganella* (r=0.63): COG0616, ENOG410Y2Y1, ENOG41126WC; *Hafnia* (r=0.63): COG0616, ENOG41126WC
P10: T-G V D N P G H P F I-M	Cleavage at N-terminal	
	Serine endopeptidases (EC 3.4.21.36; EC 3.4.21.37)	*Psychrobacter* (r=0.64): COG0466, COG0542, COG1219, COG1067, COG0265
	cysteine endopeptidases (EC 3.4.22.2; EC 3.4.22.32; EC 3.4.22.53)	*Psychrobacter* (r=0.64): ENOG4111IPD
	Aspartic endopeptidases (EC 3.4.23.1)	*Psychrobacter* (r=0.64): COG0616
	Cleavage at C-terminal	
	Serine endopeptidases (EC 3.4.21.36; EC 3.4.21.37; EC 3.4.21.67)	*Psychrobacter* (r=0.64): COG0466, COG0542, COG1219, COG1067, COG0265
